# Topological and Multivalent Effects in Glycofullerene Oligomers as EBOLA Virus Inhibitors

**DOI:** 10.3390/ijms23095083

**Published:** 2022-05-03

**Authors:** Javier Ramos-Soriano, Beatriz M. Illescas, Alfonso Pérez-Sánchez, Raquel Sánchez-Bento, Fátima Lasala, Javier Rojo, Rafael Delgado, Nazario Martín

**Affiliations:** 1 Departamento de Química Orgánica, Facultad de Química, Universidad Complutense, 28040 Madrid, Spain; fj.ramos@iiq.csic.es (J.R.-S.); alfonp01@ucm.es (A.P.-S.); rsanch06@ucm.es (R.S.-B.); nazmar@ucm.es (N.M.); 2 Laboratorio de Microbiología Molecular, Instituto de Investigación Hospital 12 de Octubre (imas12), 28041 Madrid, Spain; flasala.imas12@h12o.es; 3 Glycosystems Laboratory, Instituto de Investigaciones Químicas (IIQ), CSIC–Universidad de Sevilla, Av. Américo Vespucio 49, 41092 Seville, Spain; javier.rojo@iiq.csic.es; 4 IMDEA-Nanoscience, C/Faraday, 9, Campus de Cantoblanco, 28049 Madrid, Spain

**Keywords:** glycofullerene, click chemistry, Ebola virus, antivirals, DC-SIGN

## Abstract

The synthesis of new biocompatible antiviral materials to fight against the development of multidrug resistance is being widely explored. Due to their unique globular structure and excellent properties, [60]fullerene-based antivirals are very promising bioconjugates. In this work, fullerene derivatives with different topologies and number of glycofullerene units were synthesized by using a SPAAC copper free strategy. This procedure allowed the synthesis of compounds **1**–**3**, containing from 20 to 40 mannose units, in a very efficient manner and in short reaction times under MW irradiation. The glycoderivatives were studied in an infection assay by a pseudotyped viral particle with Ebola virus GP1. The results obtained show that these glycofullerene oligomers are efficient inhibitors of EBOV infection with IC_50_s in the nanomolar range. In particular, compound **3**, with four glycofullerene moieties, presents an outstanding relative inhibitory potency (RIP). We propose that this high RIP value stems from the appropriate topological features that efficiently interact with DC-SIGN.

## 1. Introduction

With the serious situation created by the recent outbreak of SARS-CoV-2 worldwide, the need to develop new antivirals to prevent the increasing number of multidrug-resistant pathogens was announced by the World Health Organization as a priority [1]. Moreover, innovative and long-term strategies are desired to complement the use of vaccines, usually employed when the virus is already spread. In this scenario, research on the use of carbon nanostructures as potential therapeutic materials has been intensely stimulated due to their interesting properties, namely biocompatibility, biodegradability, and antiviral activity that originates from multiple mechanisms [2]. Other advantages of using carbon nanostructures are their high surface tunability through synthetic procedures and their nanometer-sized structures which are in the appropriate range for interacting with and mimicking viruses. In particular, highly symmetric [60]fullerene derivatives are on the same scale and possess similar geometry to many icosahedral viruses [3,4,5].

In this regard, during the last years different studies have revealed important antiviral activity developed by [60]fullerene derivatives. In particular, by hexakis-adducts of [60]fullerene highly derivatized with biomolecules such as carbohydrates [6,7,8,9,10] and amino acids [11]. These hexakis-adducts present icosahedral geometry, are soluble in biological media, and are not cytotoxic. The possibility of chemical modification through the use of a Cu(I)-catalyzed 1,3-dipolar cycloaddition of azides and alkynes (CuAAC) [12,13,14] click chemistry strategy has been employed by our group and others to obtain molecular materials with up to 120 carbohydrate moieties and 13 [60]fullerene units, the so-called tridecafullerenes [6]. These derivatives have shown an IC_50_ in the subnanomolar range in the inhibition of infection by an artificial model of Ebola virus (EBOV). Although CuAAC is an efficient and robust reaction that is tolerant to a variety of conditions, it requires the use of cytotoxic copper, which has to be carefully removed after the ligation has taken place. In this regard, the use of a strain-promoted azide-alkyne cycloaddition (SPAAC) [15] reaction is advantageous as it does not require the metal catalysis. Apart from that, SPAAC prevents low yields and/or difficulties in copper removal from the obtained compounds since no further purification steps are required for the elimination of copper ions [16]. The efficient outcome of the reaction is fostered by a highly favorable enthalpic release based on the decreased ring-strain, by moving from a cyclooctyne moiety to a fused ring system with favorable angles for the sp^2^ hybridation [17]. Furthermore, the CuAAC reaction can be difficulted by the copper chelating ability of carbohydrates, especially when highly multivalent derivatives are prepared. In order to increase the multivalency of the fullerene hexakis-adducts and tridecafullerenes, we developed a copper-free SPAAC strategy [18,19] which led to the preparation of molecular tridecafullerenes appended with 360 disaccharides [9]. In this case, the use of a SPAAC reaction was required owing to the ability of α(1,2)mannobiosides to chelate the copper between the two monosaccharide units. These compounds, appended with 360 mannobiosides, showed efficient inhibitory activity against Zika (ZIKV) and Dengue (DENV) viruses in the picomolar range.

**Figure 1:**
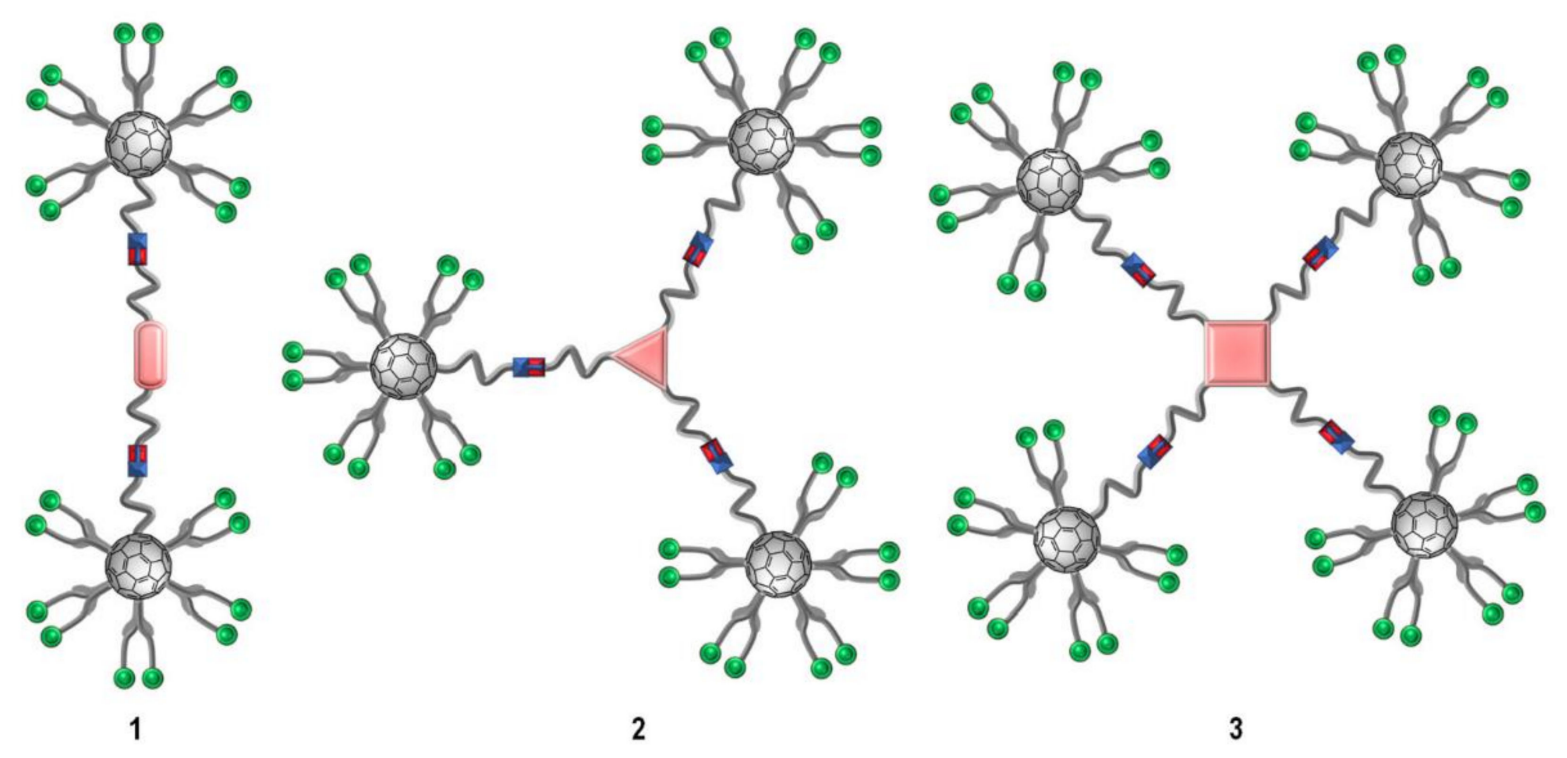
General structures of topologically different oligomers containing two, three, or four glycofullerenes (**1**–**3**).

In the aforementioned infectious viral processes of EBOV, ZIKV, and DENV, the interaction between the viral envelope and DC-SIGN (dendritic cell [DC]-specific ICAM3-grabbing nonintegrin) plays a crucial role [20]. DC-SIGN is a C-type lectin present in immature dendritic cells and on lung alveolar macrophages and is considered a universal receptor for pathogens [21]. It presents four carbohydrate recognition domains (CRDs) arranged in an almost square disposition, separated about 40 Å from each other. The CRD recognizes *N*-linked high-mannose oligosaccharides and branched fucosylated structures, and this interaction is Ca^2+^ mediated [22]. Since carbohydrate-protein interactions are generally weak, a multivalent presentation of the carbohydrates is required to increase the affinities between the glycans and their receptors. Thus, the preparation of glycomimetics with a multivalent presentation of mannoses is a strategy to block the CRDs of DC-SIGN as an antiviral target [23,24]. Therefore, continuing with our previous studies on multivalent [60]fullerene-based glycoconjugates, we focused on the synthesis and evaluation of new derivatives with different topologies and a variable number of glycofullerene moieties (Figure 1). Our interest was centered on how these factors influence the inhibitory antiviral activity of the new glycofullerene derivatives. The synthesis of these glycofullerenes relies on the grafting of a clickable A_10_B macromonomer on three different cores appended with two, three, or four cyclooctyne moieties likely to be functionalized by SPAAC click chemistry.

To obtain the clickable A_10_B asymmetric building blocks, a two-step procedure was followed, which implied the addition of two different malonates on the C_60_ surface by a Bingel-Hirsch cyclopropanation reaction leading to an asymmetric hexakis-adduct [25]. The final glycofullerene macromonomer could be employed to functionalize the different cyclooctyne containing platforms. The biological study of the cis-cell infection by EBOV in a model assay allowed us to compare the multivalent effect in the final compounds.

## 2. Results and Discussion

### 2.1. Synthesis

**Scheme 1:**
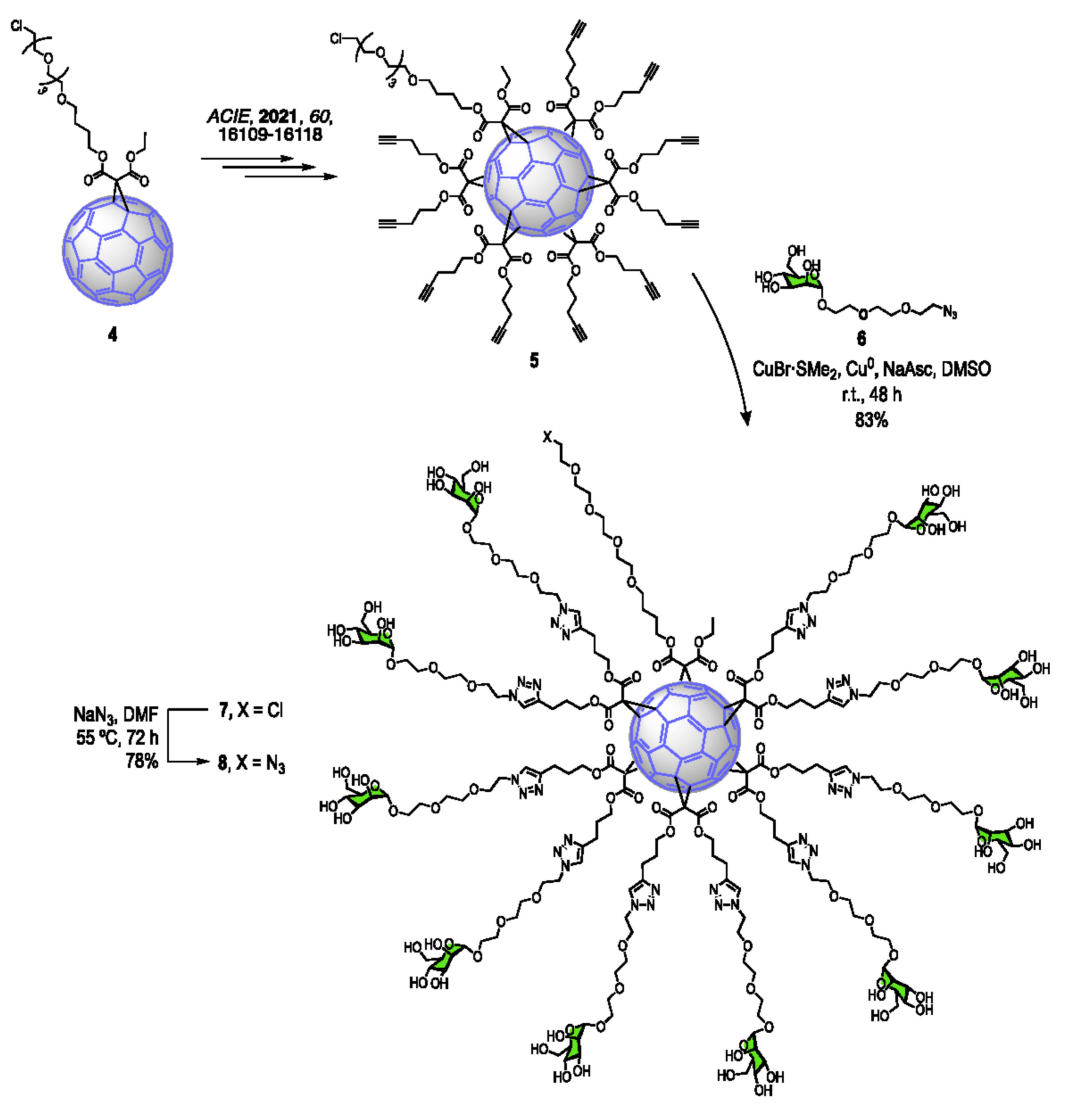
Synthetic scheme for the preparation of asymmetric glycofullerene **8**.

The synthesis of glycoconjugates **1**–**3** was carried out by following a click chemistry strategy, which required the preparation of a clickable glycofullerene containing ten mannose units and an azido group. The synthesis of this asymmetric A_10_B macromonomer started from hexakis-adduct **5** [26], as depicted in Scheme 1. Glycofullerene **7** was obtained by CuAAC addition of 2-[2-(2-azidoethoxy)ethoxy]ethyl α-D-mannopyranoside (**6**) [27] to polyalkyne **5** in DMSO, using CuBr·S(Me)_2_ as catalyst [14,28], after purification by treatment with Quadrasil MP resin to remove the copper catalyst and by size-exclusion chromatography using a Sephadex G25 gel filtration system (H_2_O:MeOH, 9:1). A simple FTIR analysis evidenced the absence of the typical bands for alkyne and azido groups (at ~2120 and 2090 cm^−1^, respectively) present in the starting materials, indicating the complete functionalization of the alkyne groups in the cycloaddition step and the efficient removal of unreacted azido-functionalised derivative **6** after purification. ^13^C NMR characterization of the obtained glycofullerene is the key to demonstrating the octahedral symmetry of the compound. Thus, only two signals appear for the sp^2^ carbons of the C_60_ cage (at ~141.3 and 145.3 ppm), together with the signal at δ ∼ 69.0 for the sp^3^ carbons of the C_60_ core, thus providing evidence of the highly *T*-symmetrical structure. In addition, only one signal is observed for the twelve equivalent carbonyl groups at δ ∼ 163.2, and two signals accounting for the twelve equivalent 1,2,3-triazole rings at ~146.1 and 122.9 ppm are observed. After treatment with sodium azide, chlorinated glycofullerene **7** allowed the quantitative preparation of the building block **8** appended with 10 sugar moieties (Scheme 1). This quantitative transformation is also perfectly followed by the ^13^C NMR analysis, as the signal of the **C**-Cl at δ∼46.0 disappears and is replaced by the signal of the **C**-N_3_ at δ∼50.5.

Owing to the ability of hydroxyl groups of carbohydrates, as well as triazole rings, for binding copper ions, we decided to employ a copper-free SPAAC strategy in the final synthetic step for the obtention of these highly multivalent systems, without the drawbacks of employing a cytotoxic metal. As outlined before, this methodology presents some advantages when compared with CuAAC. From a synthetic point of view, although both methodologies exhibit high yields, SPAAC usually takes shorter reaction times to be completed (30 min under MW) [9,19], in contrast to CuAAC which normally requires from 48 h to 72 h. This represents an important advantage when biological applications come into play. However, SPAAC also presents some disadvantages [29], namely a lack of regiospecificity, which is not usually considered as a drawback for most biological applications, including bioconjugation under reagent-free mild conditions [30,31,32,33,34,35,36].

**Scheme 2:**
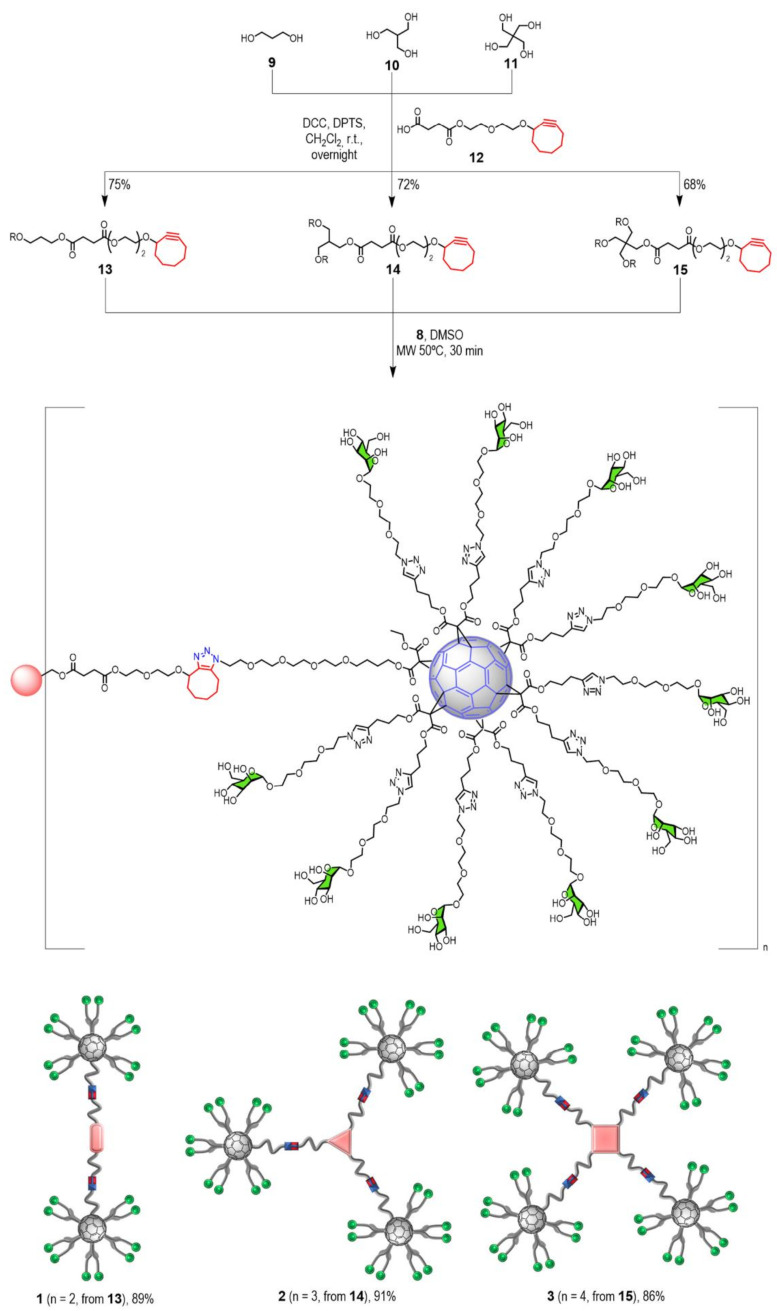
General synthetic procedure for the preparation of the central core structures **13**–**15** and subsequent SPAAC reaction for the preparation of oligomers **1**–**3**.

For this purpose, we carried out the synthesis of three different central scaffolds containing 2, 3, and 4 cyclooctyne moieties (Scheme 2). Esterification of cyclooctyne derivative **12** [18] to different commercially available alcohols with DPTS/DCC as coupling agents generated the cyclooctyne scaffolds **13**–**15** in good yields. 

Compounds **13**–**15** were completely characterized by analytical and spectroscopic techniques. The ^13^C NMR spectra present two different signals for the succinic carbonyl groups at δ ~ 172.3 and 172.0 and the two signals for the Csp of the cyclooctyne units at 100.1 and 92.7 ppm (see Supplementary Materials).

Once the central cores **13**–**15** were synthesized and fully characterized, they were submitted to the SPAAC click chemistry reaction with asymmetric glycofullerene building block **8** (Scheme 2). The addition reactions were carried out in DMSO under mild microwave irradiation (MW) conditions (50 °C), allowing the very efficient core functionalization in short times (30 min) to afford biocompatible oligomers **1**–**3** (obtained respectively from **13**–**15**). These systems were purified by size-exclusion chromatography using a Sephadex G25 gel filtration system (H_2_O:MeOH, 9:1) and subsequent filtration using Amicon^®^ Ultra-4, MWCO 10 kDa. The yields obtained were over 85% in all cases.

The structure of final multimeric glycoconjugates **1**–**3** containing 20, 30, or 40 mannose residues with two, three, or four glycofullerene moieties surrounding the central scaffold, respectively, were confirmed by FT-IR, ^1^H NMR, and ^13^C NMR (see Supplementary Materials).

**Figure 2:**
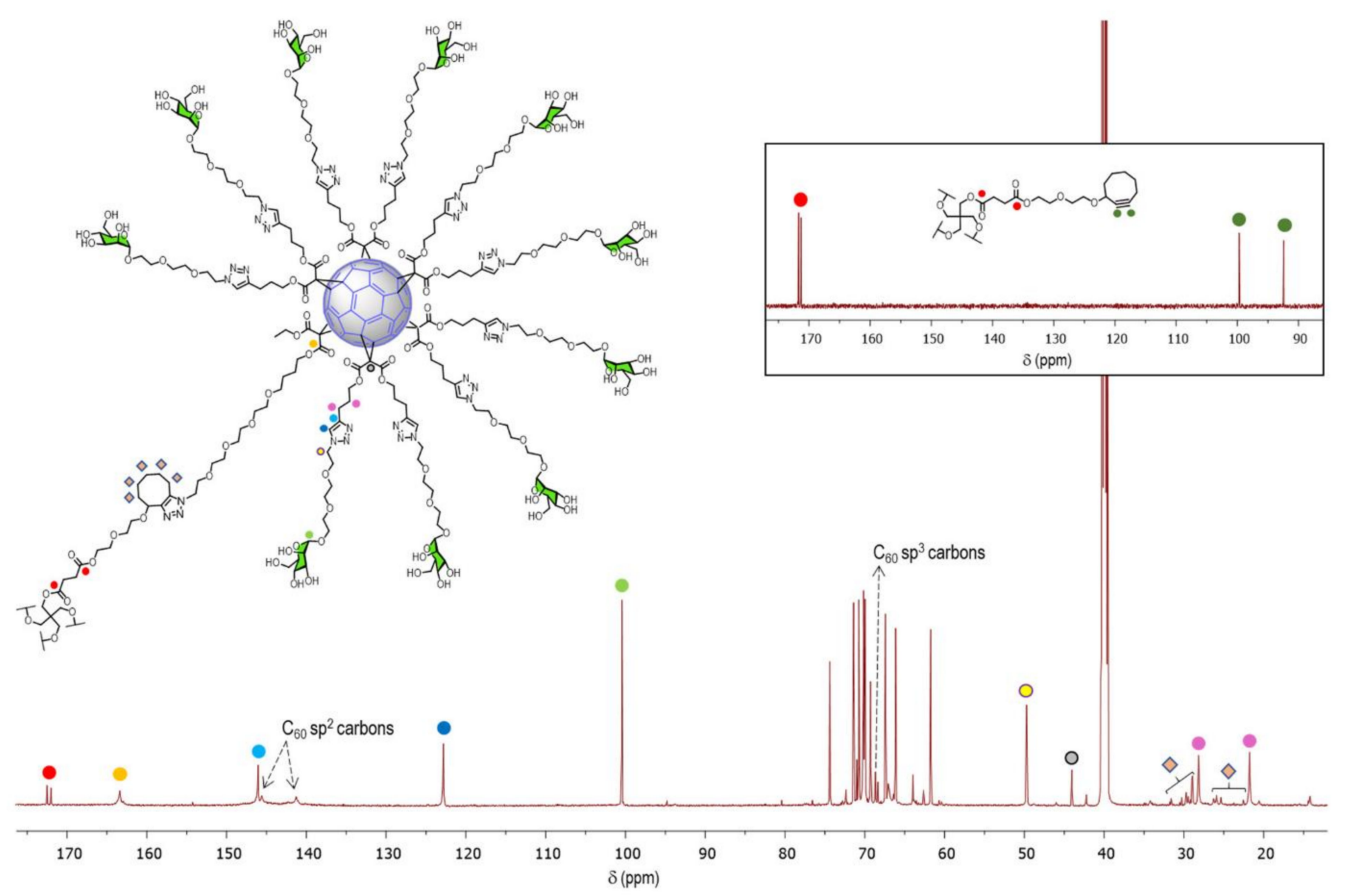
^13^C NMR of compound **3** in DMSO-*d*_6_ (175 MHz) and compound **15** (inset) in CDCl_3_ (75 MHz).

As an example, ^13^C NMR of compound **3** shows the same characteristic signals observed for the glycofullerene precursor (Figure 2). As expected, the spectrum presented only three signals for the C_60_ carbons at δ ~141.1 ppm and ~145.9 ppm for the Csp^2^, and one for the Csp^3^ at δ ~69.2 ppm. The presence of the signals of the cyclooctyne central core (δ ~20.0–35.0 ppm, ~42.2 ppm, ~171.9 ppm, and ~172.4 ppm) together with the disappearance of the resonances of the alkyne carbons allow confirmation of the proposed structure. 

It is worth noting that MS spectra (data not shown) of these kind of glycoconjugates were difficult to obtain and the molecular ion peak could not be clearly observed owing to the very high molecular mass of the compounds and high level of fragmentation arising from both the sugar residues and the Bingel-Hirsch addition pattern on the [60]fullerene sphere [6,10,37,38]. However, NMR characterization provided clear and unambiguous evidence of the structural identification of new compounds. 

### 2.2. Biological Studies

As aforementioned, DC-SIGN plays a significant role in the cell entrance of EBOV, thus facilitating early viral dissemination [20]. Therefore, DC-SIGN is considered a good model for studying the first steps of pathogenesis of EBOV and screening the antiviral activity of different compounds targeting DC-SIGN. Mannosylated and fucosylated multivalent systems are recognised by DC-SIGN and can act as virus entry inhibitors, preventing virus binding and infection of host cells. Interestingly, this antiviral strategy precludes virus mutations and resistance development [8,39].

**Figure 3:**
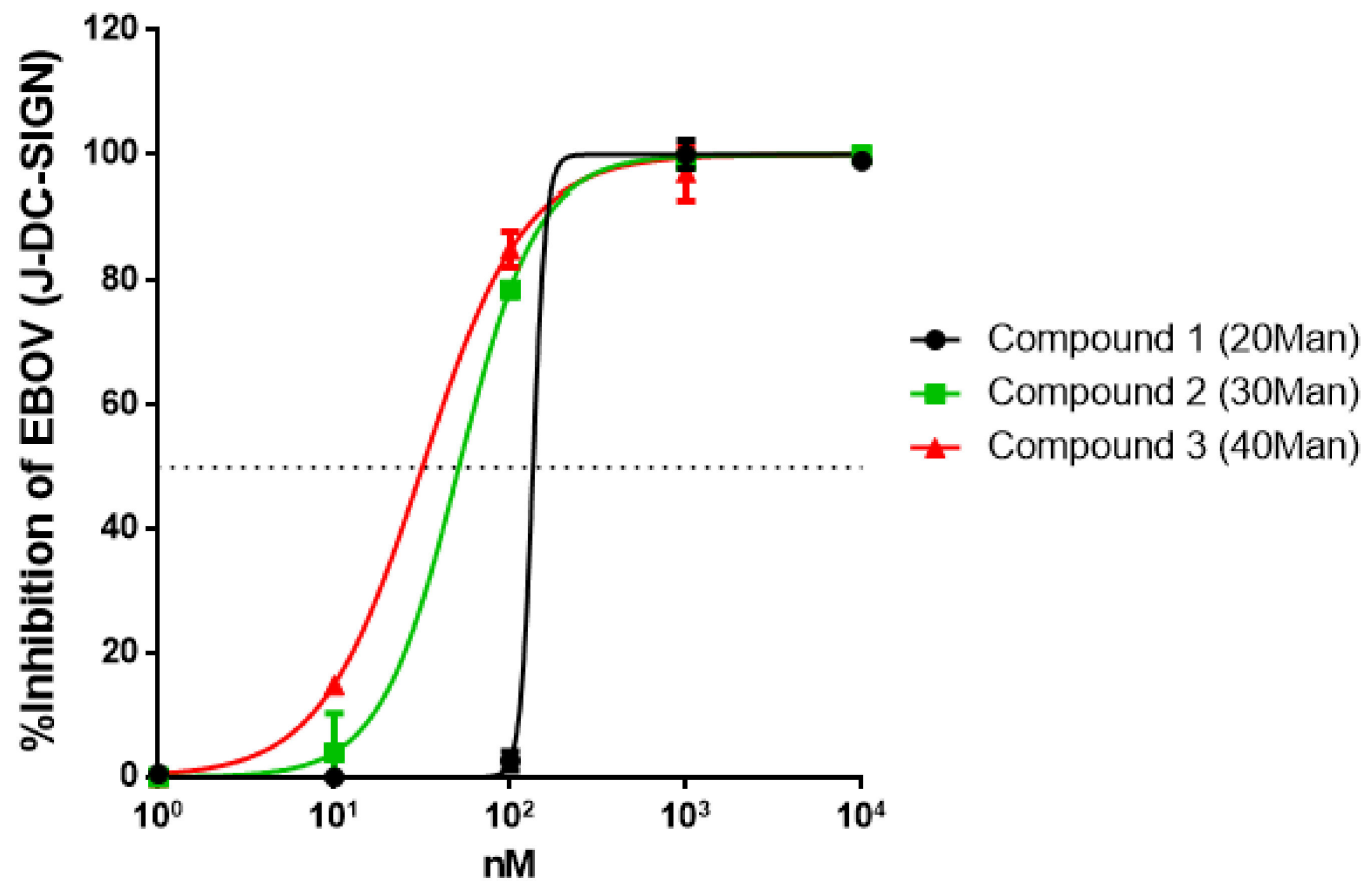
Effects of compounds **1** (20 Man), **2** (30 Man), and **3** (40 Man) on Ebola pseudotype virus infection on Jurkat DC-SIGN. Jurkat DC-SIGN cells were cultured in media containing increasing concentrations of compounds **1**, **2**, or **3** (0–10^4^ nM) for 30 min. before infection with EBOV pseudotyped viral particles. After 48 h, virus infection was measured and illustrated as percentage of inhibition with respect of the control. Values on the graph correspond to the mean of 3 independent experiments with error bars corresponding to the standard errors of the mean. The IC_50_ values were estimated using GraphPad Prism v6.0 with a 95% confidence interval and settings for normalized dose–response curves.

The activity of compounds **1**–**3** as inhibitors of EBOV infection was assessed in the experiment of cis-infection employing pseudotyped viral particles endowed with Ebola virus glycoprotein GP1. These systems show no cytotoxicity in cell lines up to a concentration of 1 μM, allowing the study of their potential biological function in preventing viral infection (Figure S1 in the Supplementary Materials). The results obtained in the infection experiments revealed the mannose-dependent inhibition effect (Figure 3). The three compounds showed high efficiency with IC_50_ values ranging from 32 to 135 nM. Compound **3**, with a higher number of glycofullerenes and 40 mannose units, shows the best affinity for the receptor. Interestingly, compounds **2** and **3** are more efficient than related glycofullerenes appended with 36 mannoses (Table 1), which can be accounted for by a topologically more favorable interaction. This observation is confirmed if we make an overall analysis of the relative potency per α-Man unit values (RIP) calculated from the existing data for monovalent α-methyl-D-mannoside [40]. The RIP value calculated for compound **3** is the higher except for the most active tridecafullerene **GF1**, which contains 13 fullerene scaffolds, a long spacer, and 120 mannose units in a globular presentation (see structure in Supplementary Materials) [6]. The value for **3** is even higher than that obtained for virus-like particles appended with up to 1620 mannose units, reported in [41]. This high relative potency can be related with the presence of four glycofullerenes that could be interacting with the tetramer cluster that forms the multivalent recognition surface of DC-SIGN. Previous studies show how the control of ligand presentation employing adequate nanoscaffolds can improve selectivity to solve crossactivity problems for many receptors [42].

## 3. Materials and Methods

### 3.1. Synthesis

***General.*** Reagents and solvents were purchased as reagent grade and used without further purification. Compounds **5**, [26] **6** [27], and **12** [18] were prepared according to previously reported procedures. For column chromatography, silica gel 60 (230–400 mesh, 0.040–0.063 mm) was purchased from E. Merck and Sephadex G25 for gel filtration from GE Healthcare, Barcelona, Spain. Thin layer chromatography (TLC) was performed on aluminum sheets coated with silica gel 60 F_254_ purchased from E. Merck, visualization by UV light. IR spectra (cm^−1^) were measured on an ATI Mattson Genesis Series FTIR instrument. NMR spectra were recorded on a Bruker AC 300, AC 500, or AC 700 with solvent peaks as reference. ^1^H and ^13^C NMR spectra were obtained for solutions in CDCl_3_ and DMSO-*d*_6_. All the assignments were confirmed by one- and two-dimensional NMR experiments (COSY, HSQC, and DEPT). MALDI-TOF-mass spectra were carried out on a Bruker BIFLEXTM matrix-assisted laser desorption time-of-flight mass spectrometer using 2-[(*E*)-3-(4-*tert*-butylphenyl)-2-methylprop-2-enylidene]propanedinitrile (DCTB) as the matrix. Electrospray ionization (ESI) mass spectra were recorded with an Esquire 6000 ESI-Ion Trap from Bruker Daltonics using CH_2_Cl_2_/MeOH as solvent system. Microwave irradiation experiments were performed using a Monowave 300 (Anton Pear) apparatus. The temperature in the sealed reaction vessel was monitored by an external surface sensor.

***Synthetic procedure to obtain glycofullerenes 7–8.***

**Compound 7.-** A round vessel that contains asymmetric hexakis-adduct of [60]fullerene 5 (68.5 mg, 0.03 mmol), 2-[2-(2-azidoethoxy)ethoxy]ethyl α-D-mannopyranoside (6) (152 mg, 0.45 mmol), CuBr·S(CH_3_)_2_ (31 mg, 0.15 mmol), and sodium ascorbate (90 mg, 0.45 mmol) in DMSO (2 mL), in the presence of a metallic copper wire, was deoxygenated under vigorous stirring for three minutes and maintained at room temperature for 48 h. After that, Quadrasil MP was added, and the mixture was stirred for 15 min. Then, it was filtered off and the reaction crude was purified by gel filtration Sephadex G25 (H_2_O/MeOH, 9:1), furnishing compound 7 as a red solid-oil (141.1 mg, 83%). ^1^H-NMR (700 MHz, DMSO- *d*_6_): *δ* 7.87 (s, 10H), 4.87–4.71 (m, 22H), 4.71–4.61 (m, 20H), 4.55–4.44 (m, 30H), 4.38–4.14 (m, 22H), 3.88–3.76 (m, 22H), 3.73–3.64 (m, 30H), 3.63–3.59 (m, 12H), 3.51–3.42 (m, 90H), 3.41–3.37 (m, 12H), 3.35–3.30 (m, 12H), 2.81–2.52 (m, 20H), 2.15–1.81 (m, 20H), 1.72–1.60 (m, 2H), 1.56–1.46 (m, 2H), 1.22–1.16 (m, 3H); ^13^C-NMR (176 MHz, DMSO-*d*_6_): *δ* 163.2, 146.1, 145.3, 141.1, 122.9, 100.4, 74.3, 71.3, 70.9, 70.7, 70.2, 70.1, 69.9, 69.2, 67.4, 66.1, 61.7, 49.8, 46.0, 44.0, 31.7, 31.6, 30.2, 29.4, 29.1, 28.1, 25.9, 25.3, 22.5, 21.8, 14.2; MALDI-ToF: *m*/*z* calculated for C_262_H_329_ClN_30_O_108_: 5658.0863; found: the high level of occurring fragmentation avoided the observation of the expected molecular ion peak. 

**Compound 8.-** To a solution of glycofullerene 7 (255 mg, 0.042 mmol) in DMF (15 mL), NaN_3_ (27 mg, 0.42 mmol) was added. The reaction mixture was heated at 55 °C for 72 h with vigorous stirring. After that, the solvent was evaporated, and the reaction crude was purified by gel filtration Sephadex G25 (H_2_O/MeOH, 9:1), furnishing compound 8 as a dark red solid-oil (187 mg, 78%). ^1^H NMR (500 MHz, DMSO-*d*_6_): *δ* 8.02–7.65 (m, 10H), 4.88–4.68 (m, 22H), 4.67–4.56 (m, 20H), 4.51–4.42 (m, 30H), 4.39–4.16 (m, 22H), 3.86–3.74 (m, 22H), 3.71–3.61 (m, 28H), 3.59 (s, 12H), 3.54–3.44 (m, 92H), 3.40–3.36 (m, 12H), 3.35 –3.30 (m, 12H), 2.85–2.57 (m, 20H), 2.12–1.83 (m, 20H), 1.74–1.62 (m, 2H), 1.57–1.47 (m, 2H), 1.26–1.17 (m, 3H); ^13^C-NMR (126 MHz, DMSO-*d*_6_): *δ* 163.5, 147.2, 146.1, 146.0, 122.7, 100.4, 74.2, 71.3, 70.7, 70.2, 70.1, 70.0, 69.9, 69.3, 67.4, 66.2, 61.6, 60.5, 50.5, 49.7, 36.3, 32.7, 32.1, 31.3, 31.2, 28.2, 22.1, 21.7, 16.1; MALDI-ToF: *m*/*z* calculated for C_262_H_329_N_33_O_108_: 5665.1267; found: the high level of occurring fragmentation avoided the observation of the expected molecular ion peak.

***General procedure to obtain compounds 13–15.-*** To a solution of the corresponding alcohol 9-10 (1 eq.), 4-(2-(2-(cyclooct-2-yn-1-yloxy)ethoxy)ethoxy)-4-oxobutanoic acid (12) (1.5 eq./alcohol unit) and DPTS (1.5 eq.) in anhydrous DCM, a solution of DCC (1.5 eq./alcohol unit) in anhydrous DCM was added. The reaction was maintained under these conditions with overnight stirring. The reaction mixture was filtered off and concentrated under reduced pressure. The crude was purified by column chromatography under the appropriate conditions. 

**Compound 13.-** Following the general procedure, using propane-1,3-diol (9) (0.01 mL, 0.18 mmol), 4-(2-(2-(cyclooct-2-yn-1-yloxy)ethoxy)ethoxy)-4-oxobutanoic acid (12) (169 mg, 0.54 mmol), DPTS (80 mg, 0.27 mmol) in anhydrous DCM (2 mL), and DCC (112 mg, 0.54 mmol) in anhydrous DCM (2 mL) and purifying by column chromatography (AcOEt), compound 13 was obtained as a colorless oil (88 mg, 75%). ^1^H NMR (300 MHz, CDCl_3_): *δ* 4.19–4.14 (m, 8H), 3.77–3.68 (m, 4H), 3.67–3.60 (m, 6H), 3.60–3.54 (m, 4H), 3.47–3.39 (m, 2H), 2.61 (s, 8H), 2.26–1.99 (m, 6H), 1.96–1.69 (m, 8H), 1.65–1.49 (m, 4H), 1.42–1.30 (m, 2H); ^13^C NMR (75 MHz, CDCl_3_): *δ* 172.5, 172.4, 100.1, 92.6, 72.7, 70.3, 68.9, 68.4, 66.3, 63.9, 60.7, 42.2, 34.2, 29.7, 29.1, 29.1, 26.3, 20.6; ESI-MS *m/z* calcd. for C_35_H_52_O_12_ [M]^+^: 664.79; found: 665.78 [M + H]^+^.

**Compound 14.-** Following the general procedure, using 2-(hydroxymethyl)propane-1,3-diol (10) (8 mg, 0.075 mmol), 4-(2-(2-(cyclooct-2-yn-1-yloxy)ethoxy)ethoxy)-4-oxobutanoic acid (12) (106 mg, 0.34 mmol), DPTS (34 mg, 0.113 mmol) in anhydrous DCM (1.5 mL), and DCC (70 mg, 0.34 mmol) in anhydrous DCM (1.5 mL) and purifying by column chromatography (Hexane/AcOEt, 1:1), compound 14 was obtained as a colorless oil (53 mg, 72%). ^1^H NMR (300 MHz, CDCl_3_): *δ* 4.24–4.16 (m, 9H), 4.11 (d, *J* = 6.0 Hz, 6H), 3.72–3.56 (m, 15H), 3.52–3.39 (m, 3H), 2.68–2.54 (m, 12H), 2.41–2.31 (m, 1H), 2.25–2.04 (m, 9H), 1.98–1.70 (m, 12H), 1.69–1.50 (m, 6H), 1.47–1.33 (m, 3H); ^13^C NMR (75 MHz, CDCl_3_): *δ* 172.2, 172.0, 100.1, 92.8, 72.8, 70.4, 69.0, 68.5, 64.0, 61.9, 42.3, 37.4, 34.3, 29.8, 29.0, 29.0, 26.4, 20.7; ESI-MS *m*/*z* calcd. for C_52_H_76_O_18_ [M]^+^: 988.50; found: 1011.63 [M + Na]^+^.

**Compound 15.-** Following the general procedure, using 2,2-bis(hydroxymethyl)propane-1,3-diol (11) (8 mg, 0.059 mmol), 4-(2-(2-(cyclooct-2-yn-1-yloxy)ethoxy)ethoxy)-4-oxobutanoic acid (12) (110 mg, 0.35 mmol), DPTS (35 mg, 0.118 mmol) in anhydrous DCM (1.5 mL), and DCC (73 mg, 0.35 mmol) in anhydrous DCM (1.5 mL) and purifying by column chromatography (Hexane/AcOEt, 1:1), compound 15 was obtained as a colorless oil (52.5 mg, 68%). ^1^H NMR (300 MHz, CDCl_3_): *δ* 4.25–4.16 (m, 12H), 4.10 (s, 8H), 3.72–3.59 (m, 20H), 3.52–3.44 (m, 4H), 2.67–2.55 (m, 16H), 2.27–2.04 (m, 12H), 1.98–1.74 (m, 16H), 1.68–1.55 (m, 8H), 1.45–1.35 (m, 4H); ^13^C NMR (75 MHz, CDCl_3_): *δ* 172.1, 171.7, 100.1, 92.8, 72.8, 70.4, 69.0, 68.5, 64.0, 62.3, 53.5, 42.3, 42.1, 34.3, 29.8, 28.9, 26.4, 20.7; ESI-MS *m*/*z* calcd. for C_69_H_100_O_24_ [M]^+^: 1312.66; found: 1335.41 [M + Na]^+^.

***General procedure for SPAAC to obtain compounds 1–3.-*** A microwave vessel that contained the hexakis-adduct of [60]fullerene derivative 8 (3–6 eq) and the corresponding core-compound 13–15 (1 eq), in DMSO (2 mL) was heated by microwave irradiation under vigorous stirring for 30 min. Then, the reaction crude was purified by gel filtration Sephadex G25 using a mixture of H_2_O/MeOH (9:1) as eluent followed by ultrafiltration with Amicon^®^ Ultra-4, MWCO 10 kDa).

**Compound 1.-** Following the general procedure and using 8 (50 mg, 0.0081 mmol) and compound 13 (1.81 mg, 2.7 µmol), compound 1 was obtained as a dark-red solid-oil (29 mg, 2.4 µmol, 89%). ^1^H-NMR (700 MHz, DMSO-*d*_6_): *δ* 7.84 (s, 20H), 4.78–4.70 (m, 44H), 4.65–4.60 (m, 28H), 4.59–4.54 (m, 24H), 4.51–4.40 (m, 74H), 4.35–4.27 (m, 24H), 3.83–3.75 (m, 50H), 3.68–3.62 (m, 62H), 3.61–3.57 (m, 40H), 3.47–3.41 (m, 106H), 3.32–3.26 (m, 42H), 2.81–2.62 (m, 40H), 2.07–1.85 (m, 42H), 1.77–1.63 (m, 15H), 1.60–1.41 (m, 13H), 1.24–1.21 (m, 6H); ^13^C-NMR (176 MHz, DMSO-*d*_6_): *δ* 166.6, 163.5, 145.9, 145.2, 143.9, 141.2, 122.8, 119.5, 100.4, 74.3, 71.4, 71.0, 70.7, 70.2, 70.1, 69.9, 69.7, 69.3, 68.7, 67.4, 66.1, 61.7, 49.7, 48.66, 44.0, 32.7, 31.5, 29.4, 28.9, 28.2, 27.5, 25.9, 25.4, 22.1, 21.8, 14.3; FT-IR (KBr, ν cm^−1^): 3369, 2937, 1735, and 1243; MALDI-ToF: *m*/*z* calculated for C_559_H_710_N_66_O_228_: 11994.5992; found: the high level of occurring fragmentation avoided the observation of the expected molecular ion peak.

**Compound 2.-** Following the general procedure and using 8 (146 mg, 0.024 mmol) and compound 14 (5.24 mg, 5.3 µmol), compound 2 was obtained as a dark-red solid-oil (86 mg, 4.8 µmol, 91%). ^1^H-NMR (700 MHz, DMSO-*d*_6_): *δ* 7.81 (s, 30H), 4.79–4.70 (m, 62H), 4.66–4.56 (m, 66H), 4.50–4.42 (m, 96H), 4.37–4.26 (m, 60H), 3.83–3.75 (m, 66H), 3.69–3.63 (m, 82H), 3.61–3.58 (m, 39H), 3.46–3.43 (m, 102H), 3.41–3.38 (m, 130H), 3.34–3.29 (m, 34H), 2.77–2.61 (m, 60H), 2.30–2.24 (m, 6H), 2.06–1.90 (m, 60H), 1.85–1.78 (m, 12H), 1.73–1.47 (m, 30H), 1.24–1.22 (m, 9H); ^13^C-NMR (176 MHz, DMSO-*d*_6_): *δ* 172.4, 172.2, 163.3, 145.9, 145.5, 141.1, 122.7, 100.4, 74.3, 72.7, 71.4, 71.0, 70.7, 70.3, 70.2, 70.1, 70.0, 69.9, 69.3, 68.7, 67.4, 67.0, 66.1, 61.7, 60.6, 49.7, 44.0, 37.3, 34.9, 31.6, 30.3, 29.5, 29.2, 28.9, 28.2, 27.2, 25.9, 25.4, 22.6, 21.8, 14.2; FT-IR (KBr, ν cm^−1^): 3373, 2957, 1738 and 124; MALDI-ToF: *m*/*z* calculated for C_838_H_1063_N_99_O_342_: 17983.8831; found: the high level of occurring fragmentation avoided the observation of the expected molecular ion peak.

**Compound 3.-** Following the general procedure and using 8 (198 mg, 0.0324 mmol) and compound 15 (4.08 mg, 5.4 µmol), compound 3 was obtained as a dark-red solid-oil (110 mg, 4.6 µmol, 86%). ^1^H-NMR (700 MHz, DMSO-*d*_6_): *δ* ^1^H NMR (700 MHz, DMSO) δ 7.82 (m, 40H), 4.82–4.70 (m, 80H), 4.66–4.60 (m, 40H), 4.60–4.55 (m, 38H), 4.51–4.40 (m, 120H), 4.38–4.30 (m, 40H), 4.22–4.17 (m, 14H), 4.14–4.09 (m, 18H), 4.07–4.03 (m, 14H), 3.83–3.74 (m, 80H), 3.70–3.62 (m, 100H), 3.62–3.56 (m, 58H), 3.52–3.44 (m, 330H), 3.34–3.28 (m, 40H), 2.79–2.58 (m, 80H), 2.26–2.17 (m, 8H), 2.17–2.10 (m, 8H), 2.07–1.87 (m, 80H), 1.86–1.79 (m, 16H), 1.77–1.47 (m, 56H), 1.24–1.21 (m, 12H); ^13^C-NMR (176 MHz, DMSO-*d*_6_): *δ* 172.4, 171.9, 163.3, 145.9, 145.5, 141.1, 122.8, 100.4, 74.4, 72.3, 71.4, 71.0, 70.7, 70.3, 70.2, 70.1, 70.0, 69.9, 69.2, 68.6, 68.3, 67.4, 67.0, 66.1, 63.9, 62.6, 61.7, 49.7, 44.0, 42.2, 34.2, 30.3, 29.7, 29.0, 28.9, 28.2, 26.3, 25.9, 25.4, 21.8, 20.6, 14.2; FT-IR (KBr, ν cm^−1^): 3375, 2948, 1740 and 1246; MALDI-ToF: *m*/*z* calculated for C_1117_H_1416_N_132_O_456_: 23973.1671; found: the high level of occurring fragmentation avoided the observation of the expected molecular ion peak.

### 3.2. Biological Assays

#### Methods

(1) Production of recombinant viruses

Recombinant viruses were produced according to a transient-transfection protocol using in 293T cells according to a transient-transfection protocol using 293T cells. The viral construction was pseudotyped with Ebolavirus (EBOV) envelope glycoprotein (GP) or vesicular stomatitis virus envelope GP (VSV-G) that expressed luciferase as a reporter of the infection. A total of 8 × 106 293T cells (producer cells) were seeded onto 10 cm plates 12–24 h before transfection and a total of 5 × 106 293T cells (producer cells) were seeded onto 10 cm plates 12–24 h before transfection, cultured in DMEM medium supplemented with 10% heat-inactivated FBS, 25 mg Gentamycin, and 2 mM L-glutamine. A few minutes before transfection, the medium was changed to 9 mL DMEM and chloroquine was added to a final concentration of 25 µM. 

Transfection contains 183 µL of 2M CaCl_2_, 500 ng of EBOV-GP or 2 µg of VSV-G, 18 µg of pNL4-3 luc, and 1300 µL of H_2_O. Next, 1.5 mL of 2xHBS (Hepes Buffer Saline) pH 7.00 was quickly added to the tubes and bubbled for 30 s. HBS/DNA solution was gently dropped onto medium. After 8 h of incubation at 37 °C with 5% CO_2_, the medium on the transfection plates was changed to 10 mL DMEM and once again, one day after transfection, to 7 mL DMEM. Transfection supernatants were harvested after 48 h, centrifuged at 1200 rpm for 10 min at RT to remove cell debris, and stored frozen at −80° C. 

Infectious titers were estimated by serial dilutions on HeLa or 293T cells and infectivity of the pseudotype virus was assessed by luciferase activity with the Steady-Glo luciferase assay system (Promega Corporation, Madison, WI).

(2) Infection in cis

Infection was performed on Jurkat cells (CD4+ T-lymphocyte cell line) expressing the receptor DC-SIGN on its surface. Since Ebola virus does not infect T-lymphocytes, its entry is absolutely dependent on DC-SIGN for infection of Jurkat cells. Cells were incubated at RT for 30 min with each compound and then challenged with an inoculum equivalent to a multiplicity of infection (MOI) of 0.05. 

Jurkat DC-SIGN+ cells (2.5 × 105) were plated into each well of a 96-well plate. Cells were incubated at RT for 30 min with each compound and then challenged with 5000 tissue culture infective doses (TCID) of recombinant viruses. After 48 h of incubation cells were washed twice with PBS and lysed for luciferase assay.

The range of concentrations tested was 0–104 nM. As a control, experiment of infection with VSV-G pseudoviruses was performed in the same conditions. Infection with VSV-G is independent of the presence of the DC-SIGN receptor.

(3) Statistical analysis

The values of percentage of inhibition of the infection presented on the graph correspond to the mean of 3 independent experiments with error bars corresponding to the standard errors of the mean. The 50% Inhibitory Concentration (IC_50_) values were estimated using GraphPad Prism v6.0 with a 95% confidence interval and settings for normalized dose-response curves.

## 4. Conclusions

A series of mannosylated [60]fullerene derivatives with different topologies and number of glycofullerene units has been synthesized. The use of a PEG spacer connecting the sugar with the fullerene scaffold in these compounds resulted in being a requirement to impart enough solubility in aqueous media for carrying out biological assays. Regarding the synthetic methodology, the use of three different cyclooctyne-derivatized platforms (**13**–**15**) has allowed the employ of a SPAAC copper-free strategy in the final synthetic step. In this way, short reaction times under MW irradiation facilitated purification steps and very good yields (from 86% to 91%). Furthermore, compounds **13**–**15** gave place to the formation of dimer to tetramer derivatives **1**–**3** appended with 20, 30, and 40 mannose units, respectively. These glycofullerenes have been characterized by standard spectroscopic techniques, showing the characteristic high symmetry (*T*_h_) inherent to hexakis-adducts of [60]fullerene. 

The study of compounds **1**–**3** as antiviral agents in an Ebola pseudotyped infection model reveals a mannose-dependent activity, with remarkable IC_50_ values in the nanomolar range (135 nM for **1**, with 20 mannose units; 51 nM for **2**, with 30 mannose units; 32 nM for **3**, with 40 mannose unis). Interestingly, the relative inhibitory potency calculated for compound **3** with 40 mannoses resulted in being the highest observed, compared with compounds **1** and **2.** This experimentally observed high RIP value of **3** has been accounted for by a more favored topology, which includes four glycofullerene units allowing a more effective interaction with the four CRDs of the tetrameric extra-cellular domain of DC-SIGN. In summary, the present study reveals how the control of ligand presentation employing adequate nanoscaffolds can improve not only the avidity of the ligands for their receptors, but also their relative selectivity, thus contributing to solving crossactivity problems for many receptors [42,43].

## Figures and Tables

**Table 1:** Comparison of IC_50_ values and relative inhibitory potency of different mannosylated multivalent compounds in inhibition studies using pseudotyped Ebola virus particles.

Compound ^a^	IC_50_ (nM)	n Mannoses	RIP ^b^	Ref.
**GF1LL ^c,d^ (120 Man)**	0.67	120	15800	6
**VLP ^e^ (1620 Man)**	0.91	1620	860	41
**VLP ^e^ (540 Man)**	9.62	540	244	41
**GF2 (120 Man)**	20.37	120	520	6
**3 (40 Man)**	32	40	992	This paper
**2 (30 Man)**	51	30	830	This paper
**1 (20 Man)**	135	20	470	This paper
**C60LL ^d^ (36 Man)**	300	36	117	10
**C60 (12 Man)**	2000	12	53	10
**C60 (36 Man)**	68000	36	0.5	10
**α-Methyl Man ^f^**	1.27 × 10^6^	1	1	40

^a^ Structures of compounds in this table are represented in the Supplementary Materials; ^b^ Relative inhibitory potency (RIP) calculated as (IC_50_)mono/IC_50_*valency; ^c^ GF: giant fullerene; ^d^ LL: long linker; ^e^ VLP: Virus-like particles; ^f^ α-methyl-D-mannopyranoside.

## Supplementary Materials

The following supporting information can be downloaded at: https://www.mdpi.com/1422-0067/23/9/5083/s1: Characterization details for compounds **1**–**3**, biological assays description and cytotoxicity assay.
