# Supplementary material for: Topological and Multivalent Effects in Glycofullerene Oligomers as EBOLA Virus Inhibitors
Source: Int J Mol Sci. 2022 May 3;23(9):5083. doi: 10.3390/ijms23095083 (PMC9131134; doi:10.3390/ijms23095083)
Supplement: Supplementary file 1 [file ijms-23-05083-s001.zip › ijms-1658236-supplementary.pdf]

Topological and multivalent effects in glycofullerene oligomers as Ebola virus inhibitors

Javier Ramos-Soriano [1], Beatriz M. Illescas [1,*], Alfonso Pérez-Sánchez [1], Raquel Sánchez-Bento [1], Fátima Lasala [2], Javier Rojo [3,*], Rafael Delgado [2,*] and Nazario Martín [1,4,*]

[1] Departamento de Química Orgánica, Facultad de Química, Universidad Complutense, 28040 Madrid, Spain

[2] Laboratorio de Microbiología Molecular, Instituto de Investigación Hospital 12 de Octubre (imas12) 28041 Madrid, Spain

[3] Glycosystems Laboratory, Instituto de Investigaciones Químicas (IIQ), CSIC – Universidad de Sevilla, Av. Américo Vespucio 49, Seville 41092 Spain

[4] IMDEA-Nanoscience, Campus Cantoblanco, 28049 Madrid, Spain

* Correspondence: beti@ucm.es; javier.rojo@iiq.csic.es; rafael.delgado@salud.madrid.org; nazmar@ucm.es

NMR Spectra of Compounds

Compound 7

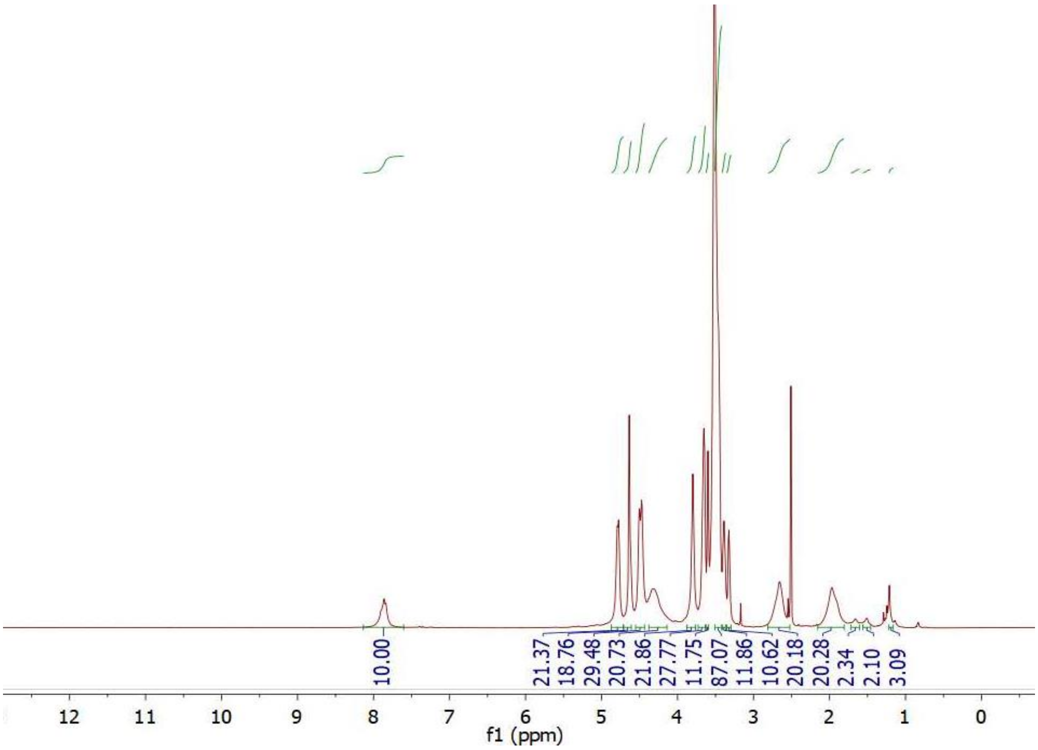

^{1}H NMR spectrum of compound **7** (700 MHz, DMSO- *d*$_6$)

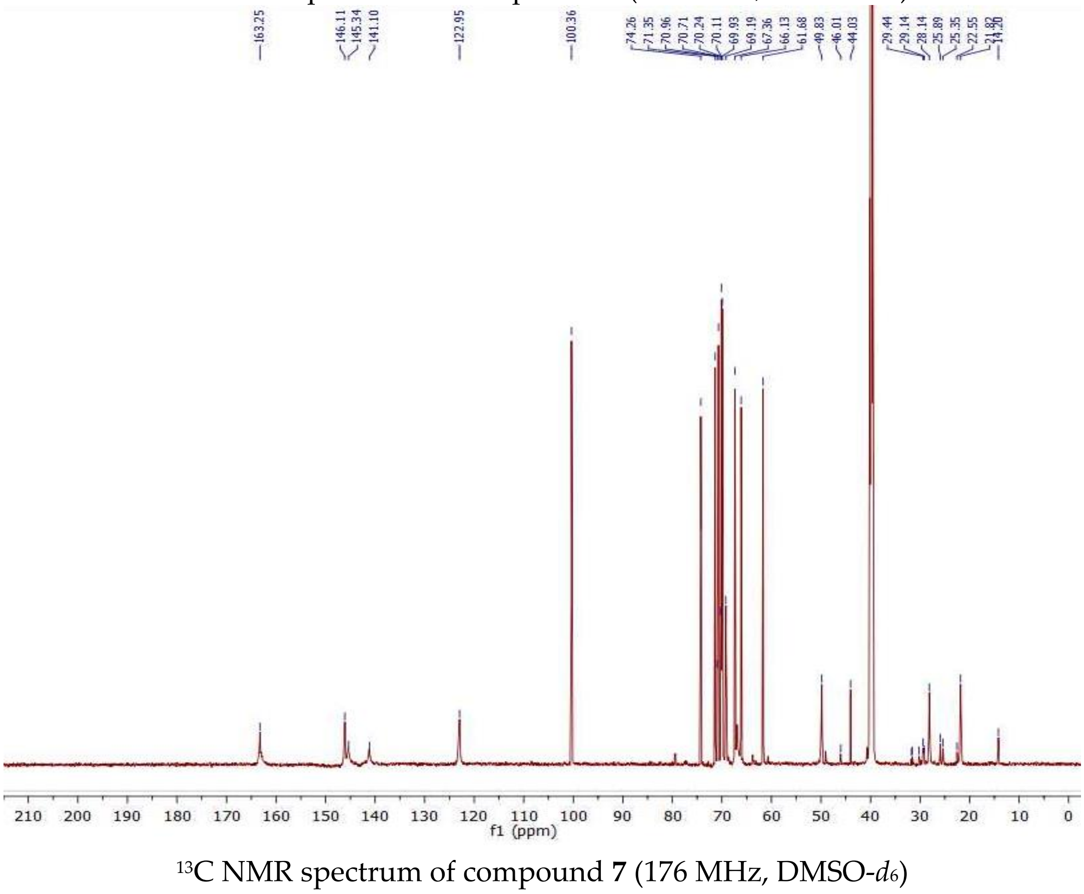

^{13}C NMR spectrum of compound **7** (176 MHz, DMSO-*d*$_6$)

Compound 8.

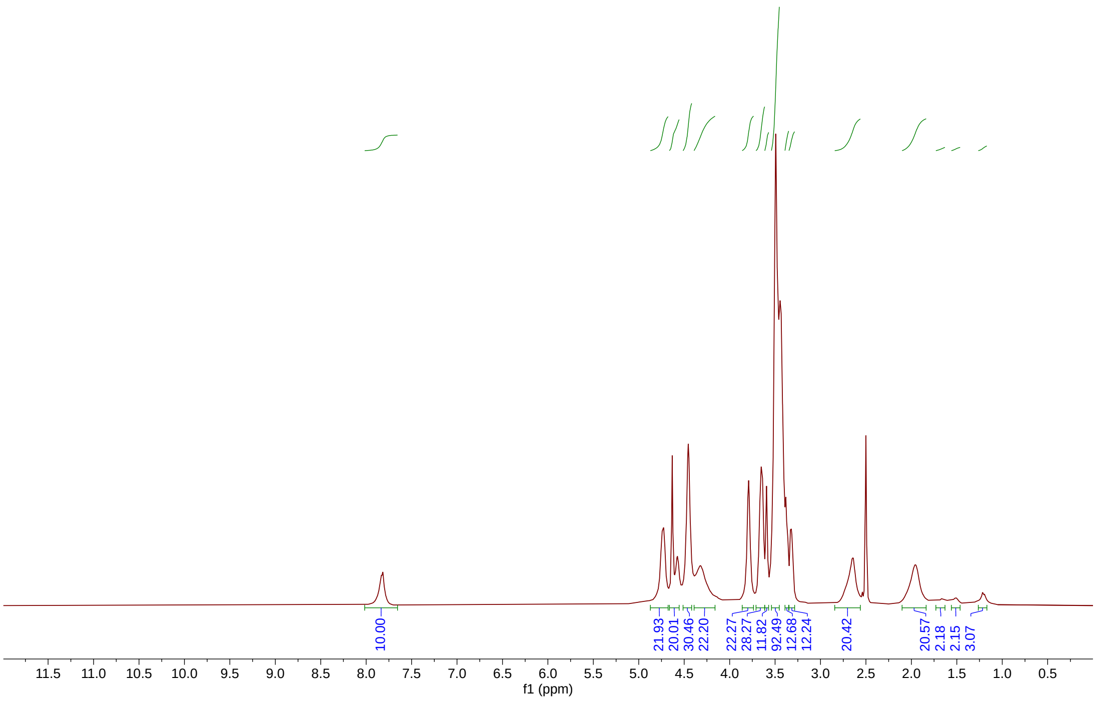

^{1}H NMR spectrum of compound **8** (500 MHz, DMSO-d_6)

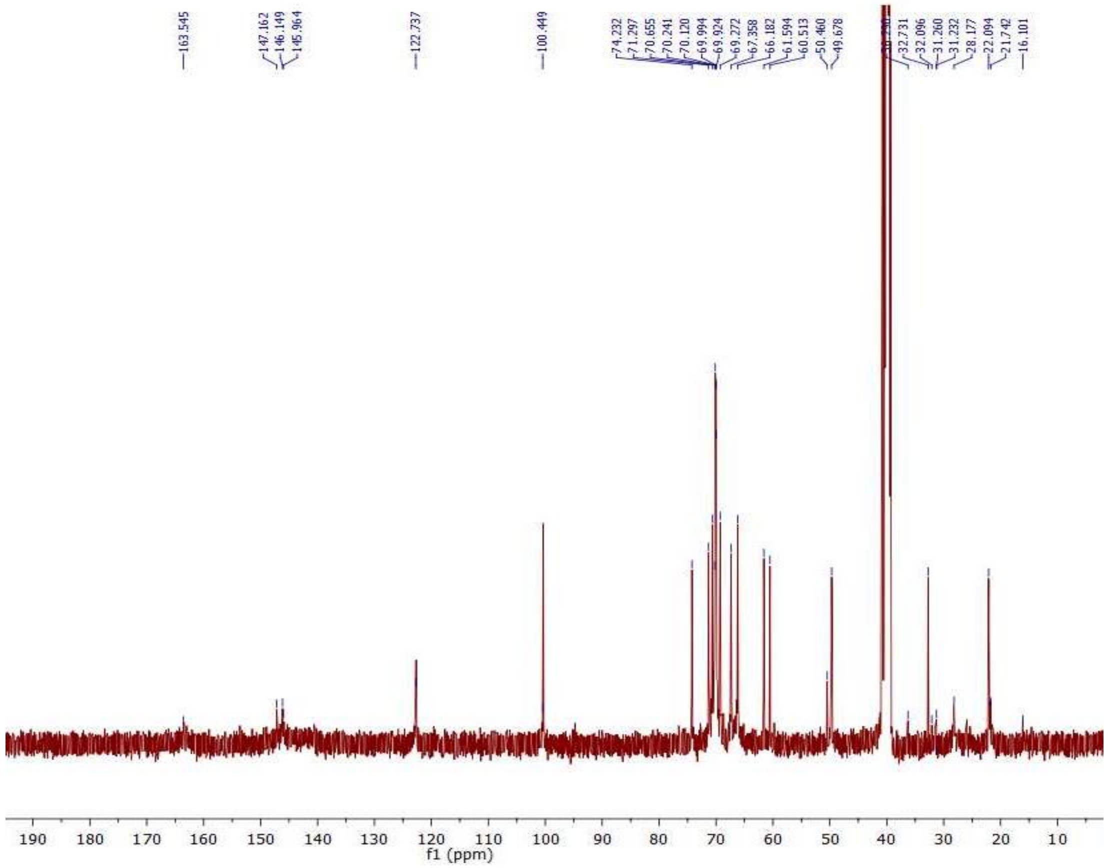

^{13}C NMR spectrum of compound **8** (126 MHz, DMSO-d_6)

Compound 13

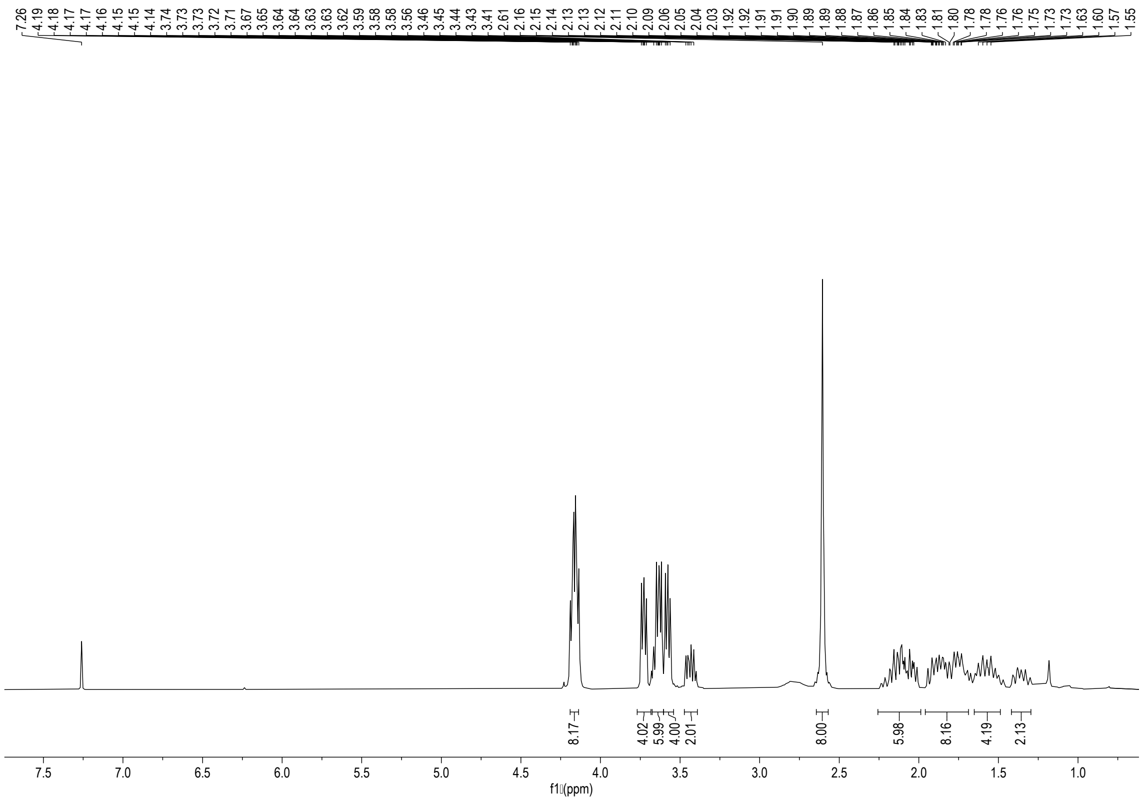

^{1}H NMR spectrum of compound **13** (300 MHz, $CDCl_3$)

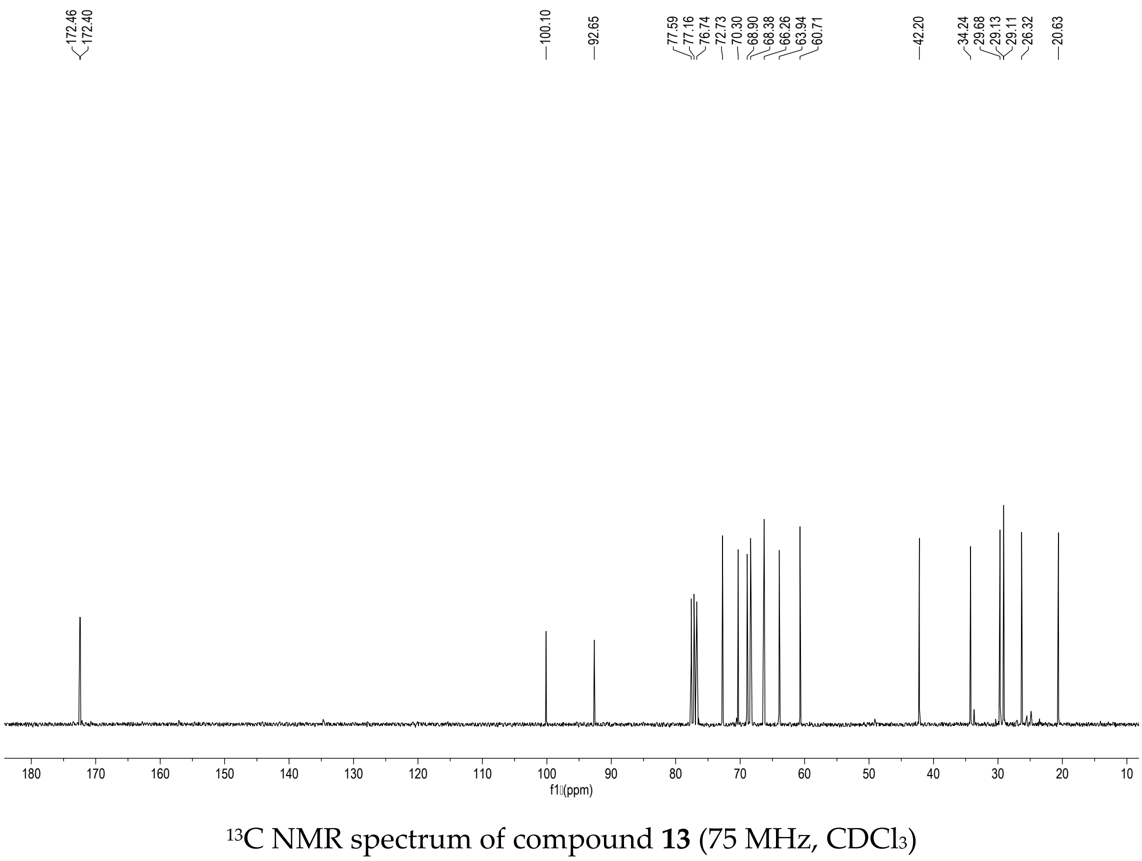

^{13}C NMR spectrum of compound **13** (75 MHz, $CDCl_3$)

Compound 14

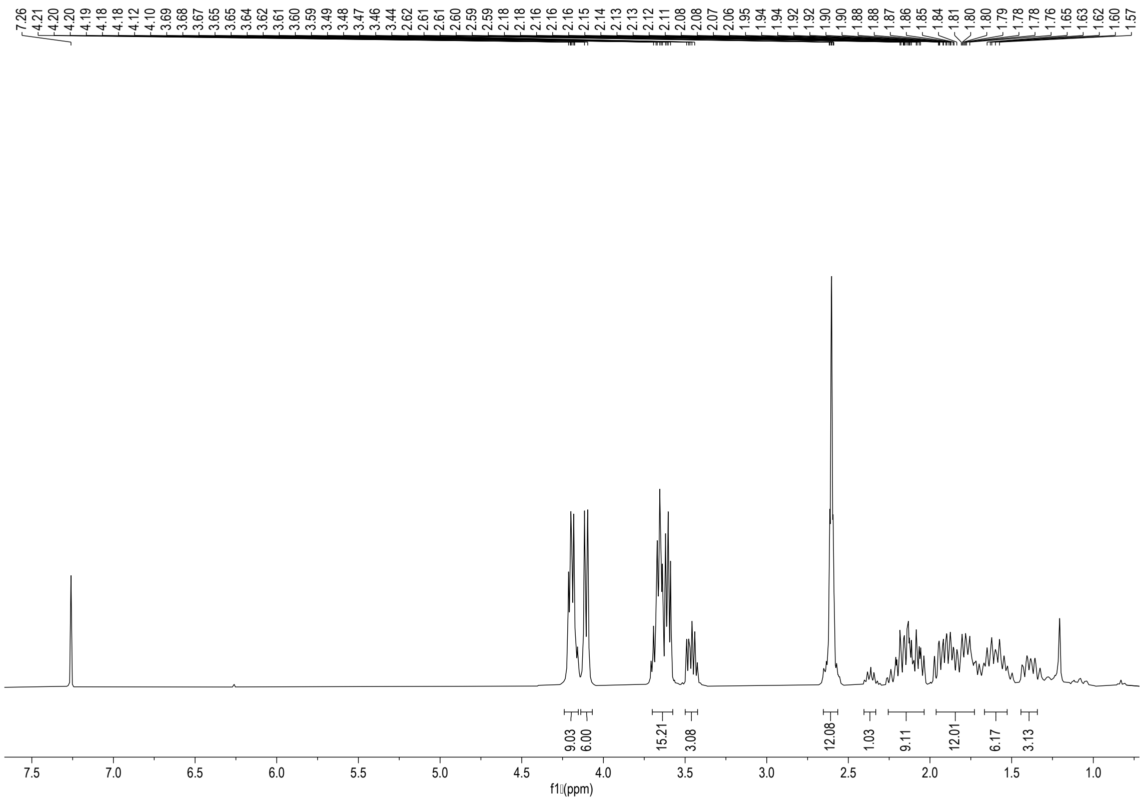

^{1}H NMR spectrum of compound **14** (300 MHz, $CDCl_3$)

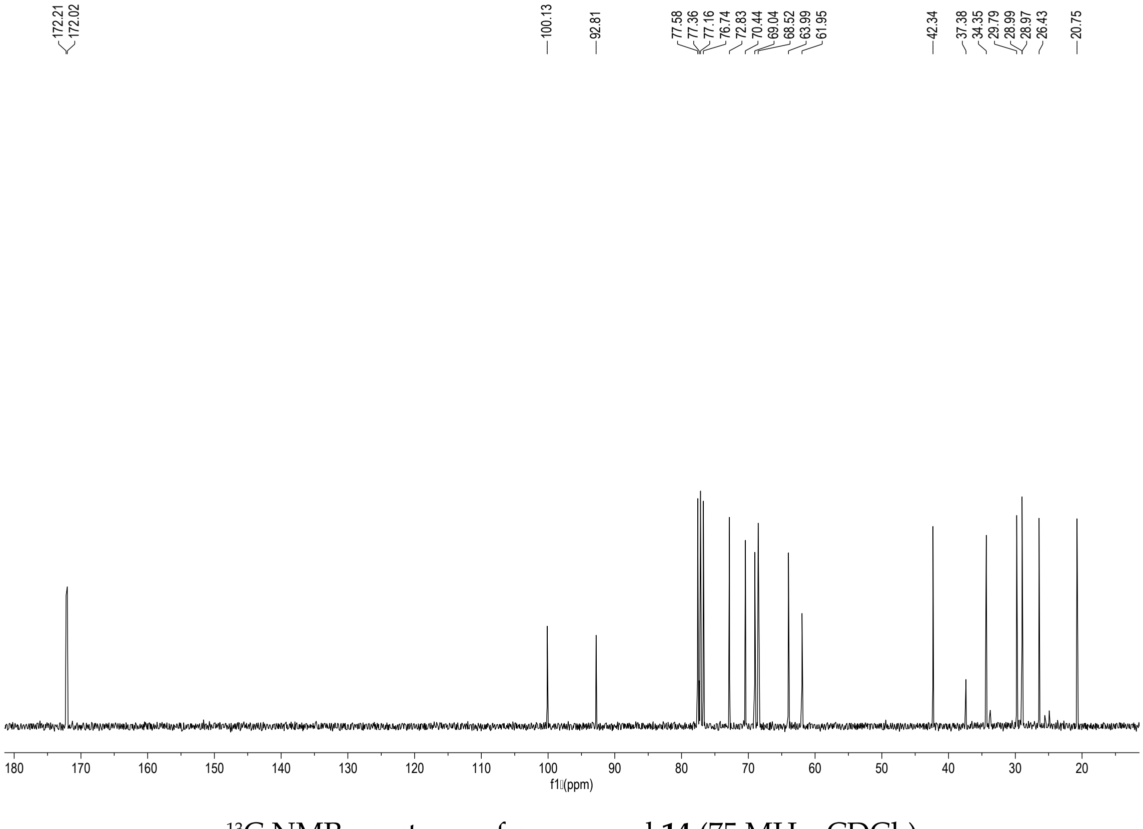

^{13}C NMR spectrum of compound **14** (75 MHz, $CDCl_3$)

Compound 15

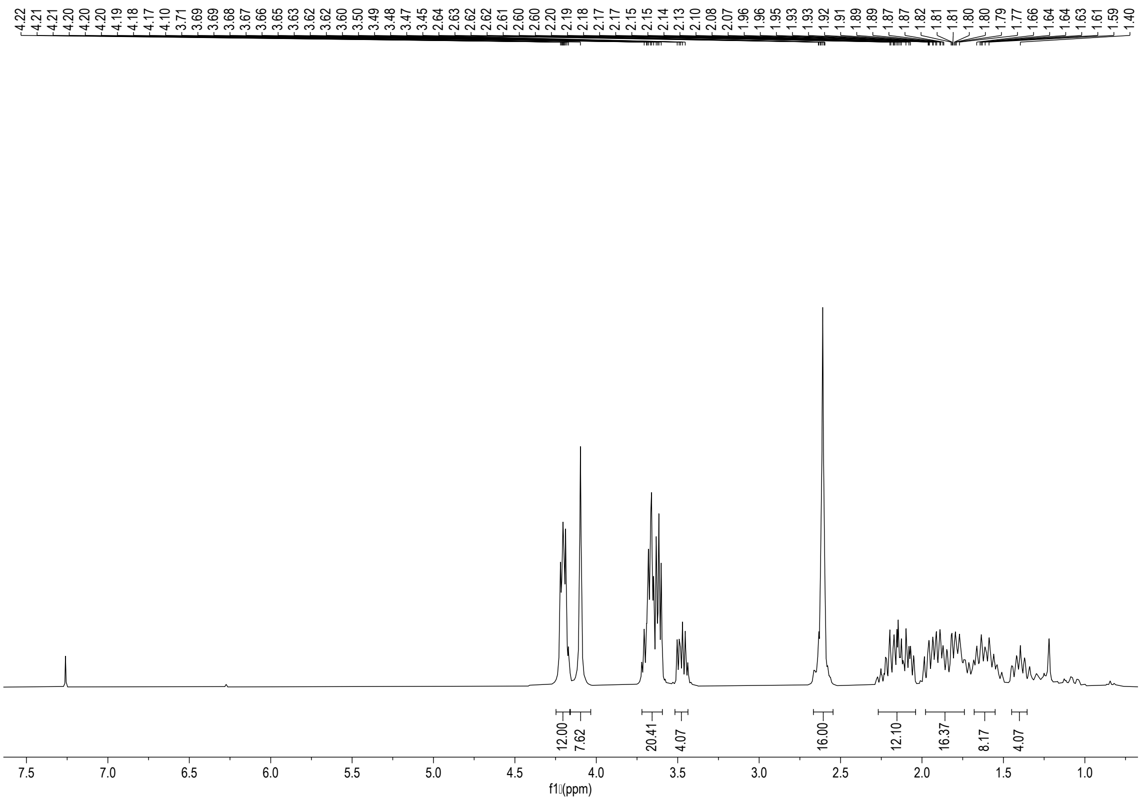

^{1}H NMR spectrum of compound **15** (300 MHz, $CDCl_3$)

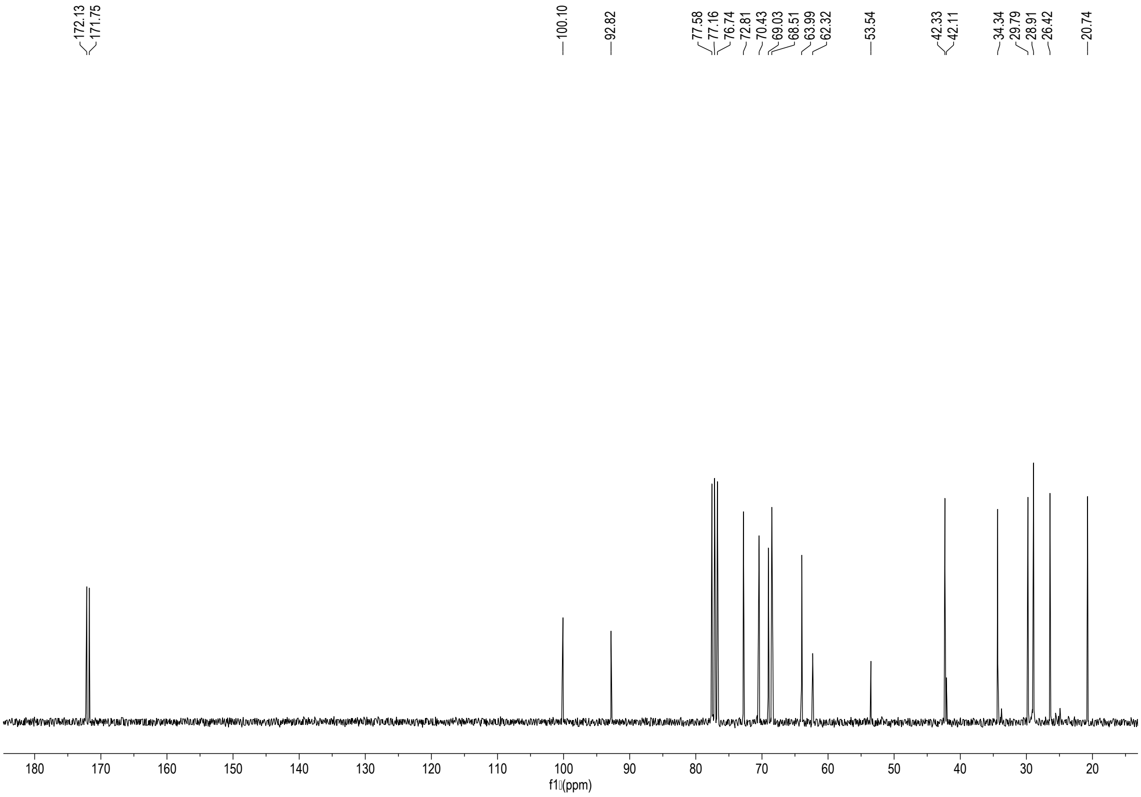

^{13}C NMR spectrum of compound **15** (75 MHz, $CDCl_3$)

Compound 1

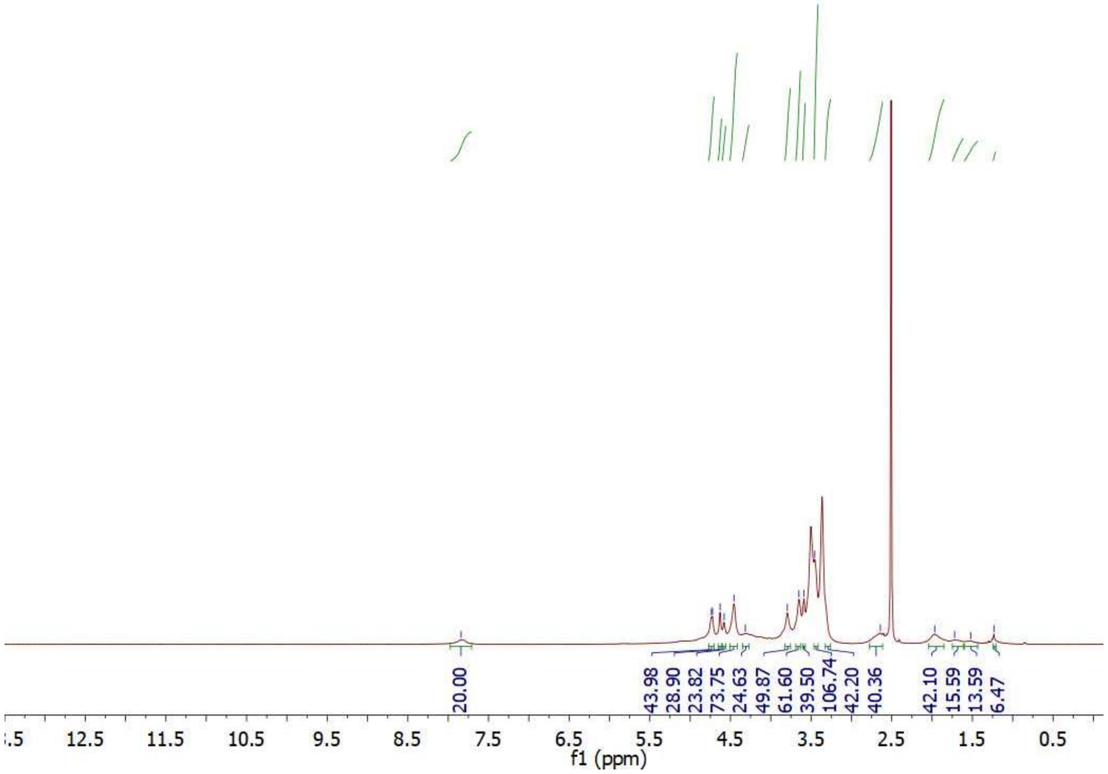

^{1}H NMR spectrum of compound **1** (700 MHz, DMSO-d_6)

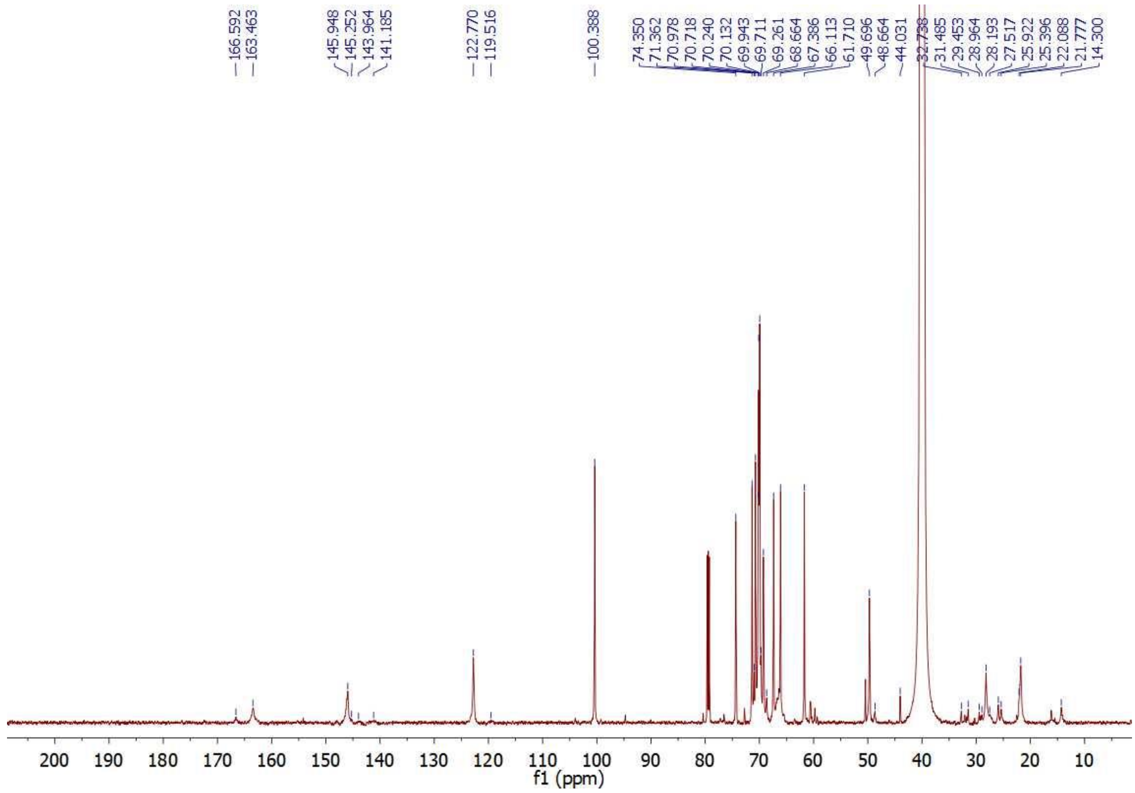

^{13}C NMR spectrum of compound **1** (176 MHz, DMSO-d_6)

Compound 2

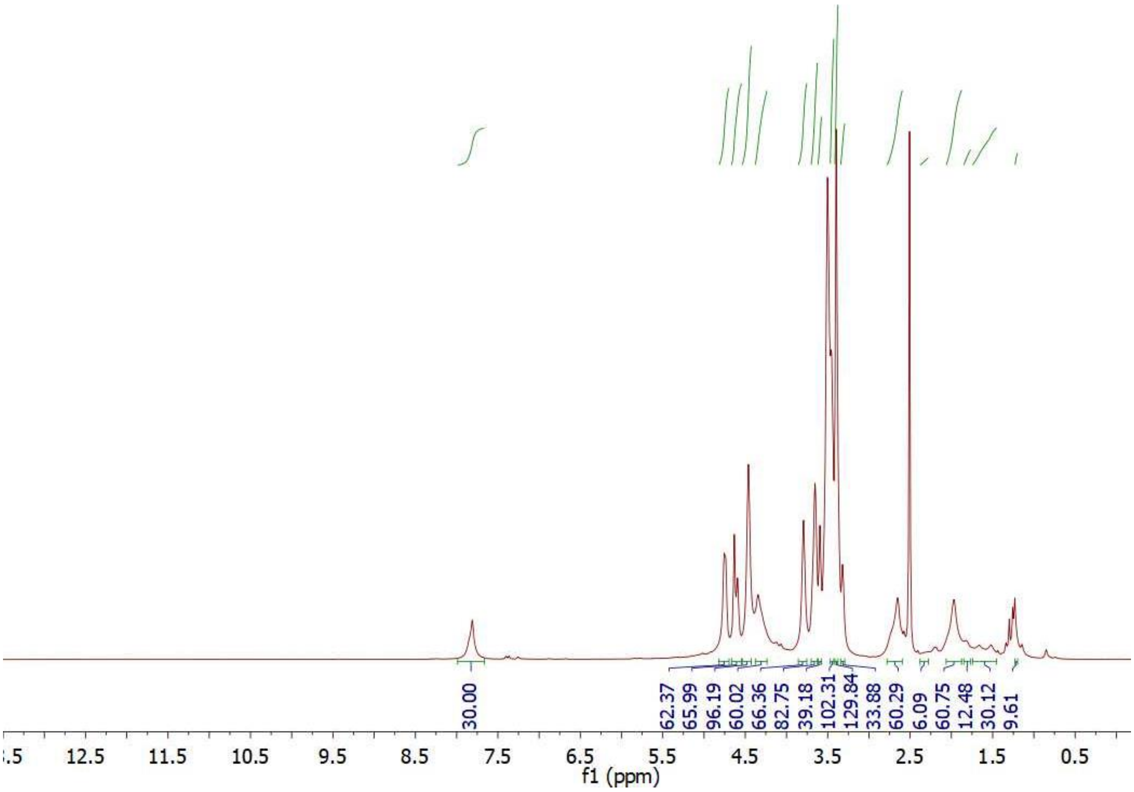

^{1}H NMR spectrum of compound **2** (700 MHz, DMSO-d_6)

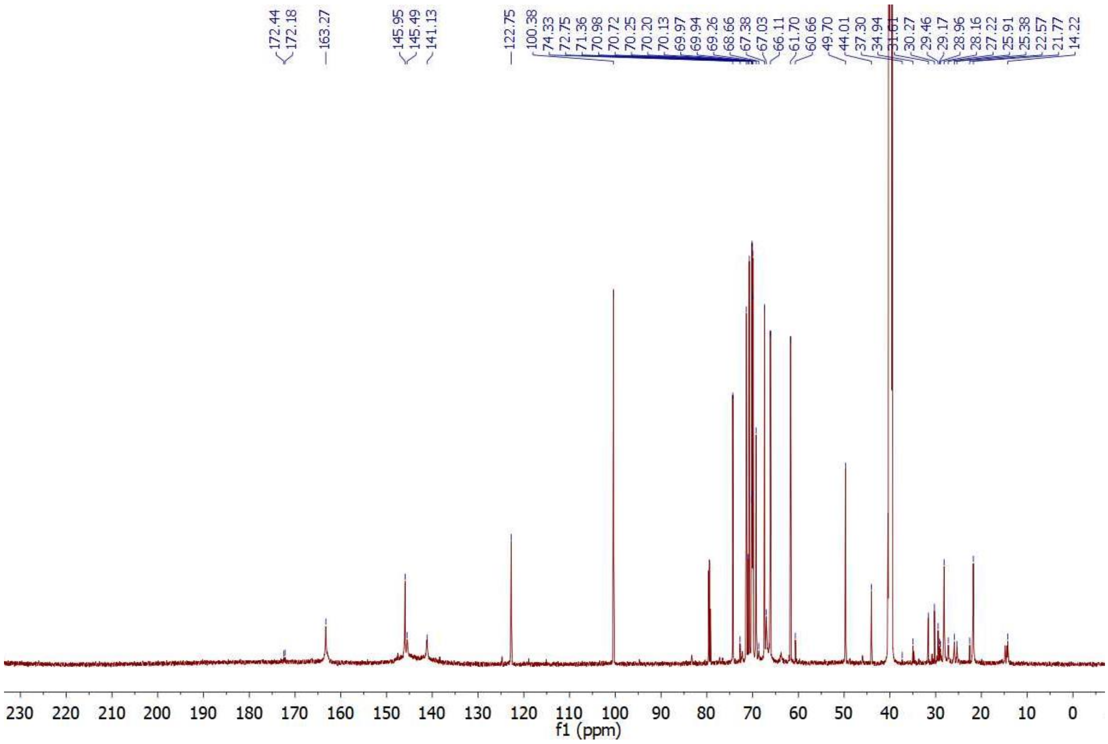

^{13}C NMR spectrum of compound **2** (176 MHz, DMSO-d_6)

Compound 3

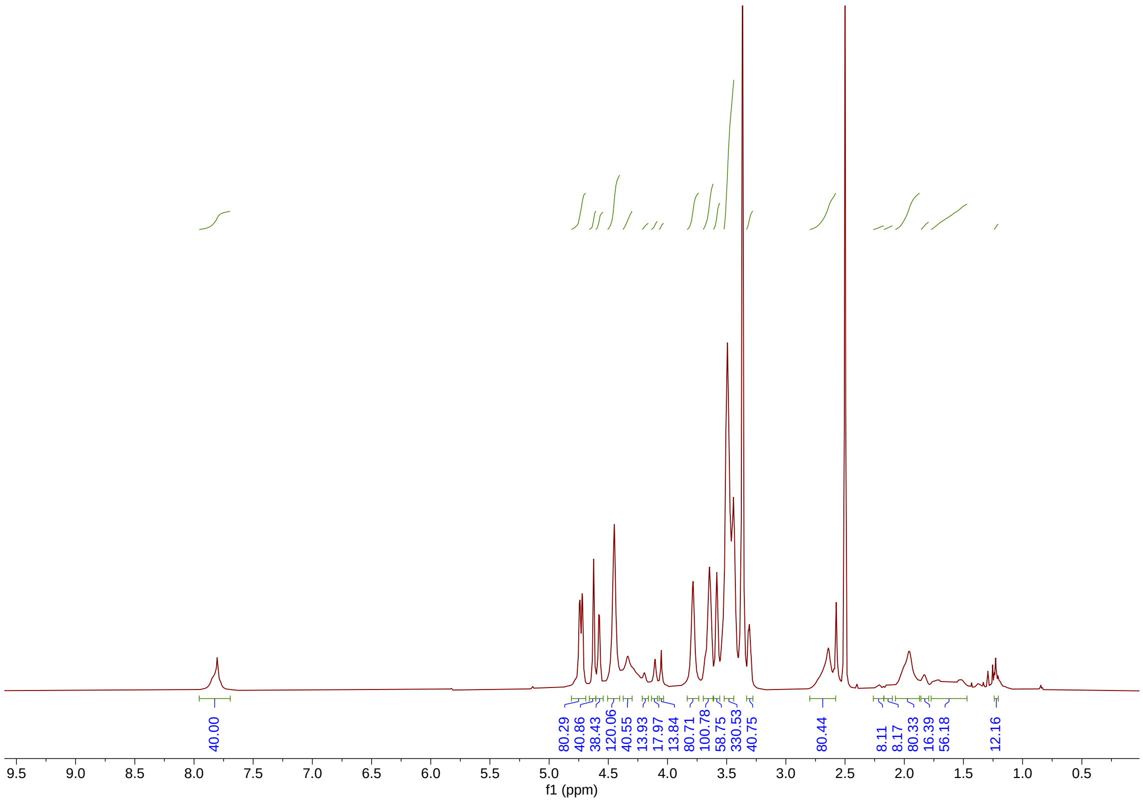

^{1}H NMR spectrum of compound **3** (700 MHz, DMSO-d_6)

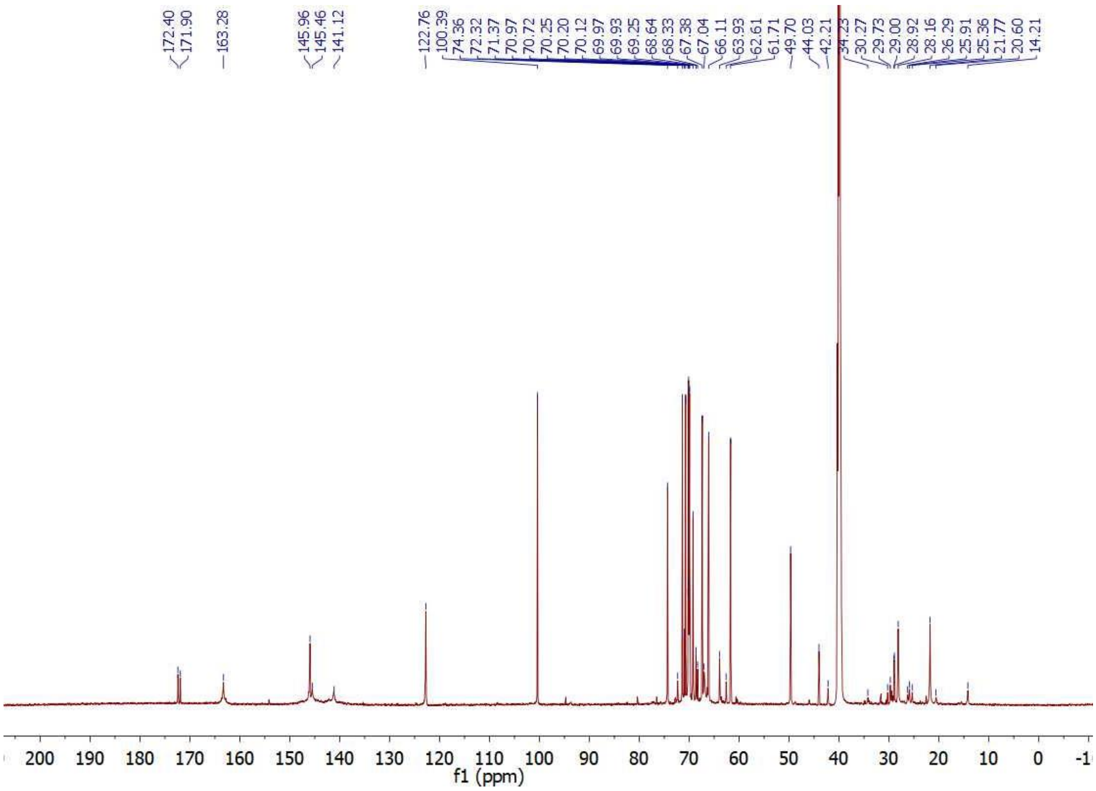

^{13}C NMR spectrum of compound **3** (176 MHz, DMSO-d_6)

Structures of Compounds in Table 1

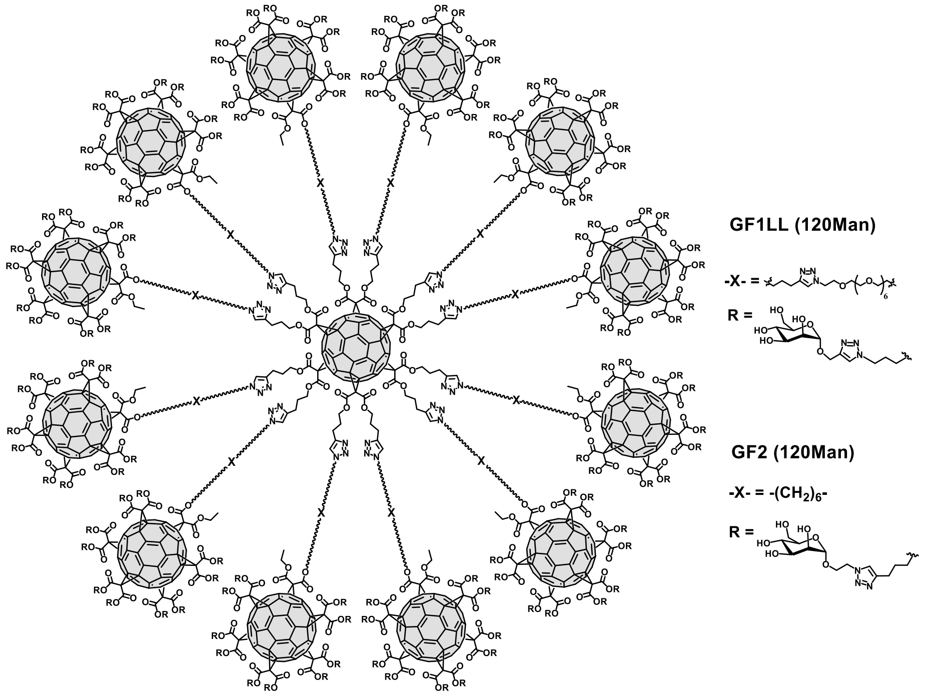

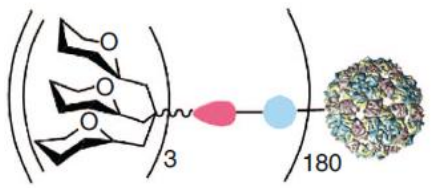

VLP (1620Man)

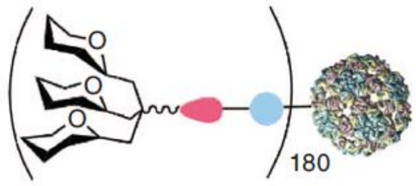

VLP (540Man)

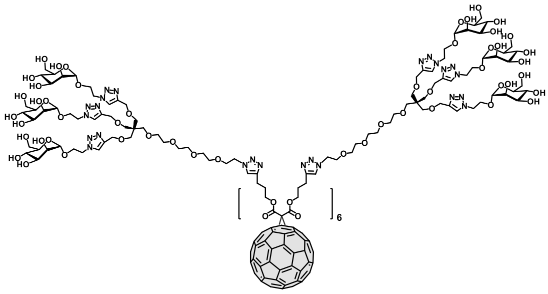

C60LL (36Man)

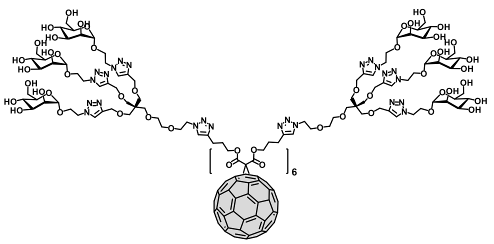

C60 (36Man)

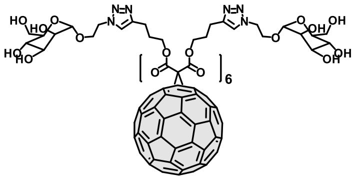

C60 (12 Man)

Cytotoxicity assay

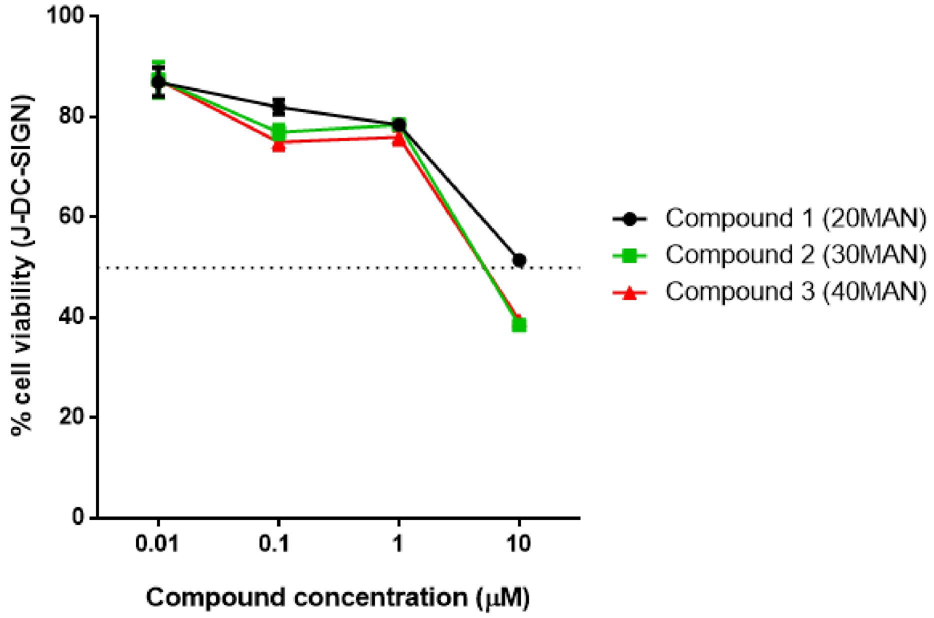

Figure S1. Cell proliferation assay using compounds **1** (20Man), **2** (30Man) and **3** (40Man). The cytotoxic effect of each compound was measured and presented as the percentage of viability of Jurkat DC-SIGN^{+} after 48h culture in the presence of different concentrations of each compound. The symbols on the graph represent the mean +/- SEM.
